# Spillover of the Atlantic bluefin tuna offspring from cages in the Adriatic Sea: A multidisciplinary approach and assessment

**DOI:** 10.1371/journal.pone.0188956

**Published:** 2017-11-30

**Authors:** Tomislav Džoić, Gordana Beg Paklar, Branka Grbec, Stjepan Ivatek-Šahdan, Barbara Zorica, Tanja Šegvić-Bubić, Vanja Čikeš Keč, Ivana Lepen Pleić, Ivona Mladineo, Leon Grubišić, Philippe Verley

**Affiliations:** 1 Physical Oceanography Laboratory, Institute of Oceanography and Fisheries, Split, Croatia; 2 Research and Development Division, Meteorological and Hydrological Service, Zagreb, Croatia; 3 Laboratory of Fisheries Science and Management of Pelagic and Demersal Resources, Institute of Oceanography and Fisheries, Split, Croatia; 4 Laboratory for Aquaculture, Institute of Oceanography and Fisheries, Split, Croatia; 5 Institute de recherché pour le developpement, UMR Botany and Modelling of Plant Architecture and Vegetation, Montpellier, France; Universita degli Studi di Bari Aldo Moro, ITALY

## Abstract

During routine monitoring of commercial purse seine catches in 2011, 87 fingerling specimens of scombrids were collected in the southern Adriatic Sea. Sequencing of the mitochondrial DNA control region locus inferred that specimens belonged to the Atlantic bluefin tuna, *Thunnus thynnus* (Linnaeus, 1758) (N = 29), bullet tuna, *Auxis rochei* (Risso, 1810) (N = 30) and little tunny, *Euthynnus alletteratus*, Rafinesque, 1810 (N = 28). According to previously published growth parameters, the age of the collected specimens was estimated at approximately 30–40 days, suggesting they might have been spawned in the Adriatic Sea, contrary to the current knowledge. A coupled modelling system with hydrodynamic (ROMS) and individual based model (IBM—Ichthyop) was set up to determine the location of the spawning event. Numerical simulations with the IBM model, both backward and forward in time, indicate commercial tuna cages in the middle Adriatic coastal area as possible spawning location. The two other non-commercial species likely opportunistically use the positive environmental (abiotic and biotic) conditions to spawn in the same area.

## Introduction

Scombridae (mackerels, tunas, and bonitos, [1]) inhabit temperate and tropical seas all around the world and carry out major feeding and spawning migrations during their life span. Their worldwide distribution and biological features are well known due to the commercial importance of these species. Along with mackerels *(Scomber colias*, *S*. *scombrus*) and bonito (*Sarda sarda*), Atlantic bluefin tuna (ABFT), *Thunnus thynnus* (Linnaeus, 1758), bullet tuna, *Auxis rochei* (Risso, 1810) and little tunny, *Euthynnus alletteratus*, Rafinesque, 1810, are widely distributed in the Mediterranean and Black Seas, as well as in the Adriatic Sea.

The Atlantic bluefin tuna (*Thunnus thynnus thynnus*, Linnaeus, 1758) is a large, highly migratory, pelagic predator displaying seasonal migrations and spawning site fidelity, in both the Mediterranean Sea and the Gulf of Mexico, which constitute its two main spawning areas [2]. Global decline of wild bluefin tuna populations as a result of heavy fishing pressure [3, 4] makes farming an attractive alternative to fisheries, although capture-based farming, a widely adopted type of bluefin production, requires a comprehensive approach to ensure sustainability. Namely, as a top predator, tuna requires large amounts of highly caloric food (herrings, sardine), which increases operational costs and space requirements, while tuna breeding in captivity, although feasible, is still hampered by various zootechnical bottlenecks, impeding its employment on a large scale [5].

In Croatia, tuna farming is based on the capture of smaller wild tunas (8–10 kg) and their subsequent farming for up to 36 months to market size (≥30 kg). Farming is performed in floating cages at sea with an annual production of up to 4,000 tonnes, exported almost entirely to the Japanese market [6]. Five semi-off shore farming locations run by four different companies are located in the central eastern Adriatic.

Considering the need for consistent tuna supply and a reduction of impacts on wild-caught juveniles, strong scientific collaboration was established in the early 2000s aimed at closing the life cycle of ABFT in Europe. Broodstock domestification and hormonal spawning induction protocols were successfully carried out, leading to the development of larval rearing technologies and formulated diets [7]. Furthermore, spontaneous spawning events of ABFT in captivity have been observed throughout the Mediterranean [8–10], including at Croatian tuna farms in the middle Adriatic where spawning behaviour was observed in the summer months [11].

Spawning of natural stocks of Atlantic bluefin tuna, bullet tuna and little tunny in Mediterranean Sea (Balearic Islands, coasts off Sicily, Libya and Cyprus) [12] occurs during the warmer part of the year; bluefin tuna from May to July [8, 13, 14], bullet tuna from June to September [14–16] and little tunny from May to September [17]. In addition to the observed homing behaviour of these spawning grounds, there is conflicting data published in non peer-review papers concerning the Adriatic Sea as a potential bluefin tuna spawning ground. New ecological niche modelling approach [13] has recognized only the central Ionian as a secondary potential spawning ground, where recent electronic tagging experiment [18] indicated this possibility. Within the same paper the Adriatic Sea was mentioned as feeding ground due to results of tagging.

Furthermore, incidental catches of tuna-like juvenile specimens, whose stocks are under strict regulation and whose spawning in the Adriatic Sea south of the island Mljet has not been previously observed, could greatly contradict our perceived knowledge on the species ecology, and may affect future decision making in stock management. Namely, 87 fingerling specimens of scombrids were collected during routine monitoring of commercial purse seine catches in September 2011 in the southern Adriatic. Morphological and molecular species identification implied the feasibility of tuna spawning in the Adriatic Sea for the first time. Upon identification of tuna and tuna-like species, the aim of this study was to determine potential spawning grounds of bluefin tuna through application of the coupled modelling system. This multidisciplinary study included a description of collected data, and results of genetic and phenotypic analyses, supported by a numerical framework of Regional Ocean Modeling System (ROMS) [19, 20] and Individual Based Model (IBM) Ichthyop [21]. Additionally, prevailing meteorological conditions and output of both models, along the comparison to available measurements, were described in details.

## Materials and methods

### Morphological and molecular identification

**Fig 1:**
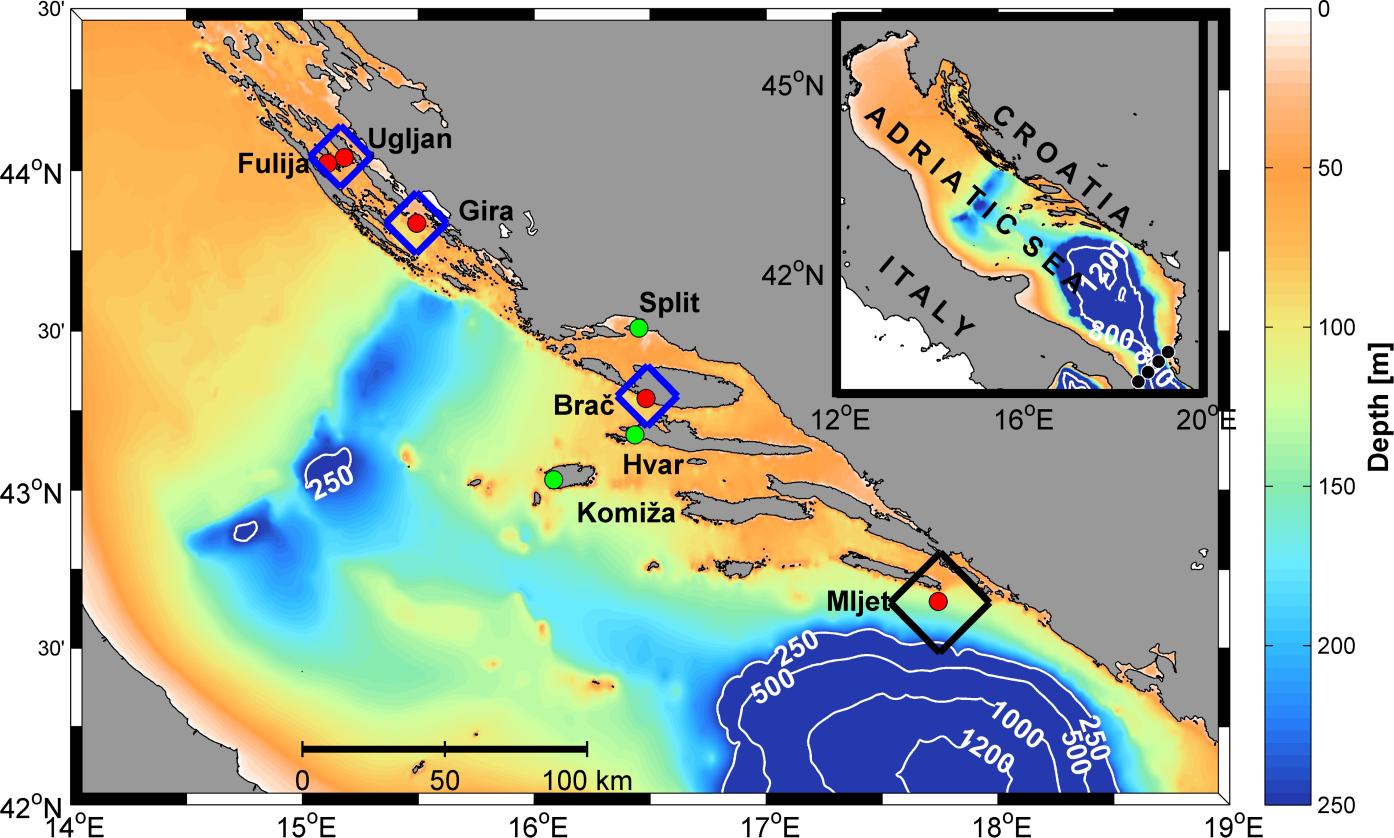
Adriatic Sea bathymetry with locations of the ABFT catch (island of Mljet) and fish cages (near islands of Ugljan (44°2'27.00''N, 15°10'48.45''E), Fulija (44°1’23.00”N, 15°6'31.55''E), Gira (43°50'11.37''N, 15°29'34.54''E) and Brač (43°17'19.93''N, 16°28'53.23''E)). Contours are drawn for 250, 500, 1000 and 1200 m depths. Blue squares are depicted around bluefin farming cages, representing the area of success for particles in the backward experiment. The black square is depicted around the natural environment of catch location, representing the area of success for particles in the forward experiment. Green dots represent coastal sea surface temperature stations (Split, Hvar, Komiža). Four black dots at the map of the entire Adriatic (upper right corner) represent points of release in the Strait of Otranto (40°11'46.32''N, 18°35'7.8''E; 40°41'20.76''N, 19°13'58.44''E; 40°31'48.36''N, 19°1'24.96' E and 40°21'18.72''N, 18°47'37.68''E) (Experiment 10, Table 1).

Unidentified scombrid specimens (N = 87) were collected on 1 September 2011 by authors of this article during routine monitoring of commercial purse seine catches along the eastern Adriatic Sea, at the Croatian fishing ground in the open sea area off coast of the island Mljet (42°38’55.32”N, 17°44’31.00”E) (Fig 1). This monitoring was conducted as part of the PERIMON project, financed by the Croatian Ministry of Agriculture, Department for Fisheries. Sampling was conducted on commercial species, which are not endangered or protected, with common fishing gear and grounds, and therefore no specific permits were required. Furthermore, animals collected from commercial fisheries with methodology normally used in fishery practices were not exposed to any unnecessary pain, injuries or suffering. Collected specimens came from the same haul and were immediately frozen, fin clips stored in absolute alcohol for molecular identification and transported to the laboratory for subsequent analysis. In the laboratory total length (*TL*), fork length (*FL*) (±0.1 mm) and total body weight (*W*) (±0.01 g) of each fish were measured (S1 Table).

#### Molecular identification

Genomic DNA was extracted from 10–50 mg of tissue using the DNeasy Tissue Kit (Qiagen Inc.) following the manufacturer’s protocol. A partial fragment of the mitochondrial DNA control region was amplified following Grubišić et al. 2013 [11]. Products sequencing were performed by Macrogen Inc. (Seul, Korea) on an ABI 3730 automatic sequencer. The obtained sequences (~800 bp) were compiled along sequences of other scombrids available in GenBank (*T*. *thynnus* AB106300.1, *A*. *rochei* AB107063.1, *E*. *alletteratus* AB099716.1) in MEGA 6 and aligned incorporated by Clustal X 1.83 [B] using default parameters. DnaSP 5.19 software [22] was applied to calculate haplotype diversity. Phylogenetic relationships among mtDNA haplotypes of scombrids were reconstructed using Neighbour-Joining analyses and p-distance calculation in MEGA 6. A bootstrap test with 1000 iteration values was performed on the unrooted NJ tree.

#### Morphometric traits

For each species, conversions between length measures were accomplished with linear regression models using the least squares method, while the length-weight relationships were calculated by applying standard exponential regression equation. In order to obtain the age of the collected specimens previously published growth parameters were used as follows:

- Atlantic bluefin tuna, *Thunnus thynnus* (Linnaeus, 1758): *FL* = 41.20+2.37*t* (*FL*:fork length in mm, *t*:age in days; [23]),
- bullet tuna, *Auxis rochei* (Risso, 1810): *FL* = 29.74(1-e^(-10)(*t*-0.018)^) (*FL*: fork length in mm, *t*:age in years; [24]),
- little tunny, *Euthynnus alletteratus*, Rafinesque, 1810: *FL* = 2.036+0.396*t* (*FL*: fork length in mm, *t*: age in days; [25]).

### Description of the modelling system

In order to determine the possible location of the spawning event, a coupled modelling system ROMS—Ichthyop was set up and run. Hourly averaged current, temperature and salinity fields were calculated using the Regional Ocean Modeling System (ROMS) [19, 20] for the period from 17 July to 2 September 2011. Detailed description of the ROMS model, its set up and evaluation of the results are given in S1 Appendix. Surface circulation of the Ionian Sea, used in the discussion, was assessed from the physical reanalysis component of the Mediterranean Forecasting System which was obtained from the Copernicus Marine Environment Monitoring Service database.

Ichthyop v.3.3.a (http://www.ichthyop.org/downloads; [21]) is a tool that uses physical (temperature, salinity, currents) and biological (egg buoyancy, diel vertical migration, swimming, larvae growth, mortality related to temperature, etc.) parameters to give an estimate on icthyoplankton dynamics. The time step in the IBM simulations was 600 seconds, and bouncing coastline behaviour was applied. The numerical Runge-Kutta 4th order scheme was used for advection. Both forward and backward simulations were performed to gain more information regarding particle dispersion and to determine possible spawning location. Decision to use 20000 particles as an optimal particle number in experiments was based on the statistic stability criterion [26]. Passive particle displacement determined by modelled currents and variable diffusivity fields was used in backward simulations with no additional options.

The starting point for simulations backwards in time was near Mljet, where particles were released from the stain with a radius of 500 m and thickness of 15 m in depth (Experiment 1, see Table 1). Simulations lasted 46 days back in time after particles were released on 1 September. Additional depth ranges based on diel larvae distribution studied near the Balearic Islands [14] and thermocline level (S1 Appendix) were tested too (Experiments 2, 3 and 4). In Experiment 4 thermocline level is used as a minimum expected depth because at age of collected specimens, ABFT is an active predator and prey could be below the thermocline. As it was assumed that collected small tuna spawned in the fish farm, sources of particles for forward simulations were the fish farms near Ugljan (this also represented the farm at Fulija due to their proximity which placed them both within the same numerical cell), Gira and Brač (Fig 1). The stain radius was 250 m to encompass the area similar to cages, while its vertical extension was within the first 15 m where most tuna are located during spawning cycles, although the cages protrude to a depth of 30 m. Each day between 17 July and 5 August at 0300 UTC 1000 particles were released from each cage location. Sensitivity experiments indicate that the use of any other release time during tuna spawning hours between 0000 and 0300 UTC [9], apart from the selected ending time, has no significant impact on dispersion results. Forward experiments were performed with passive particle displacement only (Experiment 5), with passive displacement combined with active swimming (Experiments 6 and 7) and with active swimming only (Experiments 8 and 9). Particle swimming velocity was one, two or four body lengths per second [27, 28], where body length was a function of age based on the previously stated published growth parameters. Swimming direction was randomly chosen in each time step. Two possibilities regarding swimming were used: constant speed implies that particles swim at the velocity defined as a function of age, while randomly chosen speed means that particles swim at a random speed ranging from zero to the velocity defined as a function of age. As the incubation period for eggs in captivity is 30 hours [11], this phase was neglected in the 48-day numerical experiments, and the assumption that particles had the initial length of newly hatched larvae and possibility to swim was used. An additional forward experiment (Experiment 10) was performed to test the pathways of particles originating from four points at the Strait of Otranto (Fig 1).

**Table 1:** List of numerical experiments.

Experiment number	Orientation in time	Depth of release (m)	Point of release	Passive particle movements (advection plus diffusion)	Swimming (none, constant speed, randomly chosen speed)
Experiment 1	Backward	0–15	Mljet	Yes	None
Experiment 2	Backward	15–30	Mljet	Yes	None
Experiment 3	Backward	30–50	Mljet	Yes	None
Experiment 4	Backward	20–50	Mljet	Yes	None
Experiment 5	Forward	0–15	Cages	Yes	None
Experiment 6	Forward	0–15	Cages	Yes	Constant speed
Experiment 7	Forward	0–15	Cages	Yes	Randomly chosen speed
Experiment 8	Forward	0–15	Cages	No	Constant speed
Experiment 9	Forward	0–15	Cages	No	Randomly chosen speed
Experiment 10	Forward	0–50	Strait of Otranto	Yes	None

## Results

### Molecular identification and growth performance

**Fig 2:**
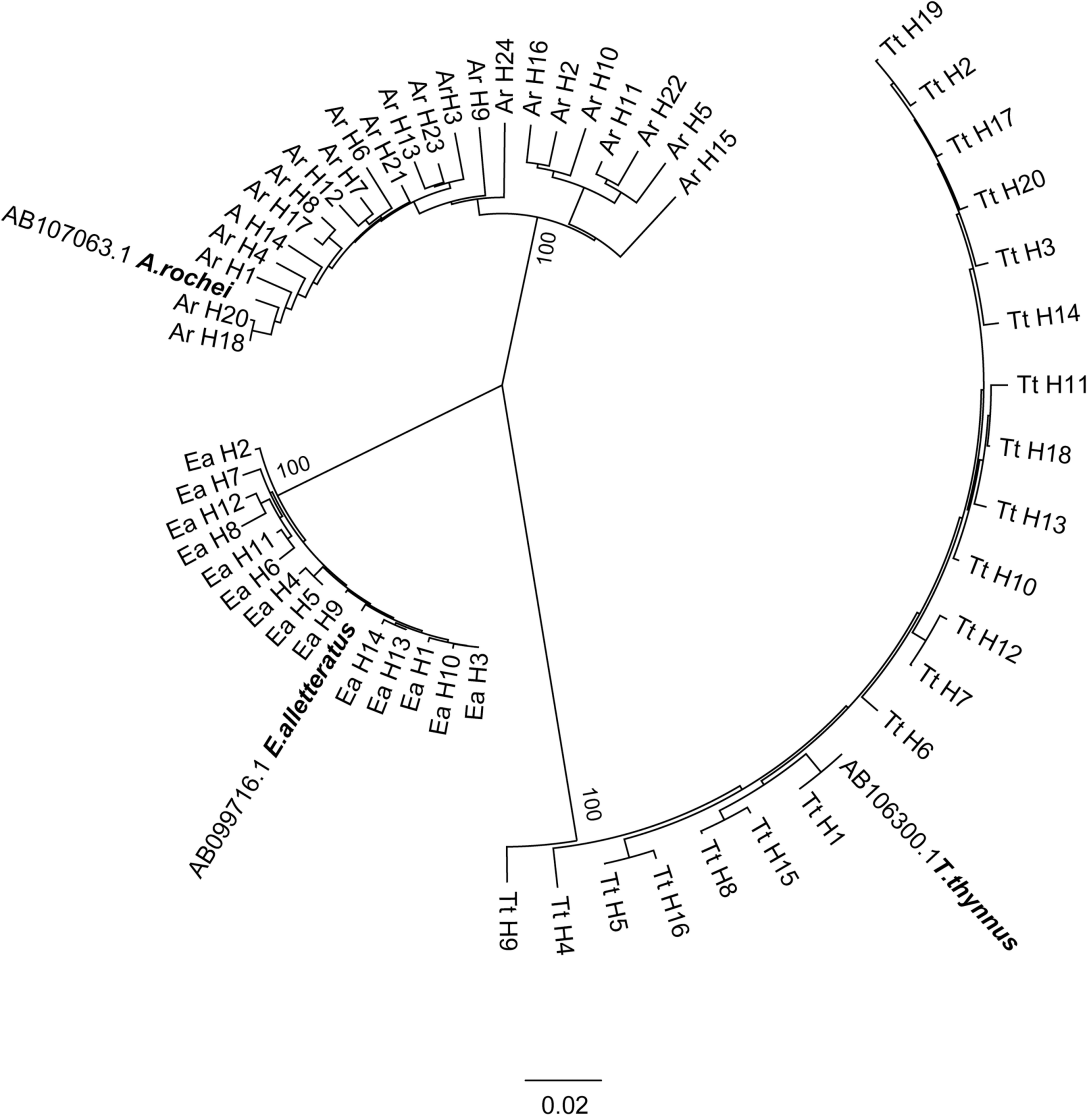
Unrooted NJ tree based on the p-distance of the mtDNA control region sequences used. Bootstrap values are shown along the branch for each clade. Haplotypes (H) of *Thunnus thynnus* (Tt), *Auxis rochei* (Ar), *Euthynnus alletteratus* (Ea) are presented.

Sequencing of the mitochondrial control region locus inferred that specimens belonged to the Atlantic bluefin tuna, *Thunnus thynnus* (Linnaeus, 1758) (*N* = 29), bullet tuna, *Auxis rochei* (Risso, 1810) (*N* = 30) and little tunny, *Euthynnus alletteratus*, Rafinesque, 1810 (*N* = 28). Namely, phylogenetic reconstruction showed the presence of three distinct clades (Fig 2) corresponding to the presence of these three scombrid species. *Thunnus thynnus* was presented with 20 haplotypes (GenBank acc. no. KY491009 –KY491028), *Auxis rochei* with 23 haplotypes (KY446395—KY446417) and *Euthynnus alletteratus* with 14 haplotypes (KY446418—KY446431).

**Fig 3:**
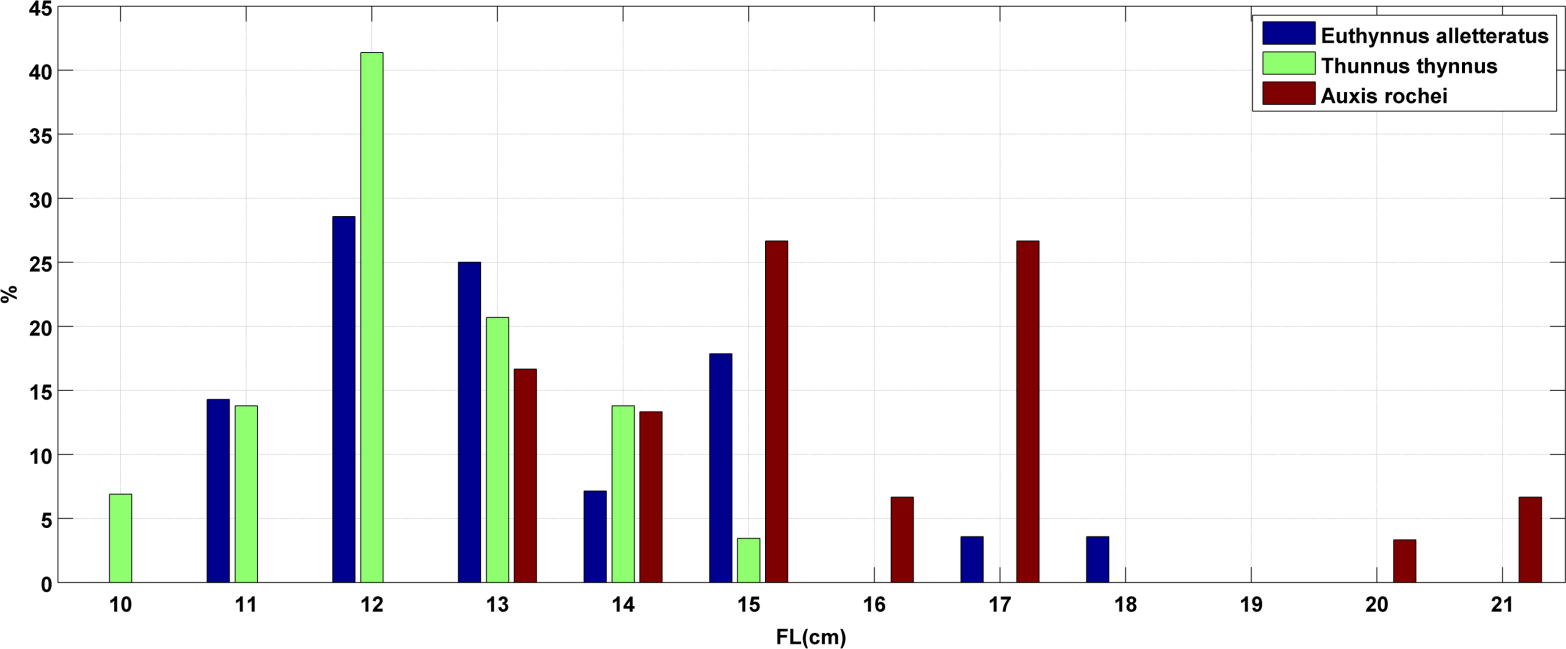
Length frequency distribution of Atlantic bluefin tuna, *Thunnus thynnus* (Linnaeus, 1758), bullet tuna, *Auxis rochei* (Risso, 1810) and little tunny, *Euthynnus alletteratus*, Rafinesque, 1810 collected with commercial purse seiners operating in the southeastern Adriatic, September 2011.

Length frequency distribution of each analysed scombrid species is given in Fig 3. Fork length range of Atlantic bluefin tuna specimens was somewhat narrower (10.8<*FL*<15.0 cm; mean±*SD*: 12.7±1.09 cm) than for bullet (13.2<*FL*<21.8 cm; mean±*SD*: 16.1±2.27 cm) and little tuna (11.6<*FL*<18.4 cm; mean±*SD*: 13.6±1.73 cm). Accordingly, the measurable range and mean total body weight of Atlantic bluefin (16.97<*W*<53.45 g; mean±*SD*: 30.74±9.356 g) was lower than for bullet (23.83<*W*<148.88 g; mean±*SD*:55.74±31.837 g) and little tuna (12.94<*W*<93.21 g; mean±*SD*:35.45±18.465 g).

Conversions between total (*TL*) and fork (*FL*) length and the length-weight relationship for each studied species are given in Table 2. All obtained length–length regressions and length-weight relationships were statistically significant (*P* < 0.001) with high values of the correlation coefficient (0.967 ≤ *r* ≤ 0.999). Statistically significant positive allometric growth was established for all three species (Atlantic bluefin tuna: *t*-test = 241.6, *df* = 28, *P*<0.0001; bullet tuna: *t*-test = 82.7, *df* = 27, *P*<0.0001; little tunny: *t*-test = 88.6, *df* = 29, *P*<0.0001).

**Table 2:** The length–length (between total (*TL*) and fork (*FL*) length) and length-weight (fork length (*FL*)–total body weight (*W*)) relationships separately given for each scombrid species, Adriatic Sea, September 2011.

Species	TL-FL	FL-W
Atlantic bluefin tuna	*FL* = 0.9424*TL*-0.456	*W* = 0.0039*FL*^3.5152^
Bullet tuna	*FL* = 0.9517*TL*-0.016	*W* = 0.0035*FL*^3.4435^
Little tuna	*FL* = 0.9283*TL*+0.164	*W* = 0.0019*FL*^3.7387^

According to previously published growth parameters, the age of each scombrid species was estimated (S1 Fig). In general, the overall scombrid age range was between 14.8 (bullet tuna *FL* = 13.2 cm) and 45.9 (Atlantic bluefin tuna *FL* = 15 cm) days, while the average age of all studied specimens was 29.5±7.02 days.

### Meteorological and oceanographic conditions

**Fig 4:**
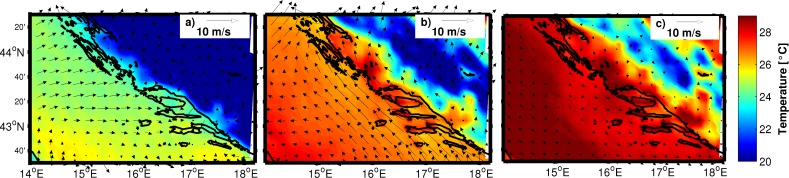
**Daily mean of air temperature at 2 m and wind field at 10 m height obtained from ALADIN on 22 July (a), 8 August (b) and 25 August (c).** Wind vectors are plotted at every ninth grid point.

**Fig 5:**
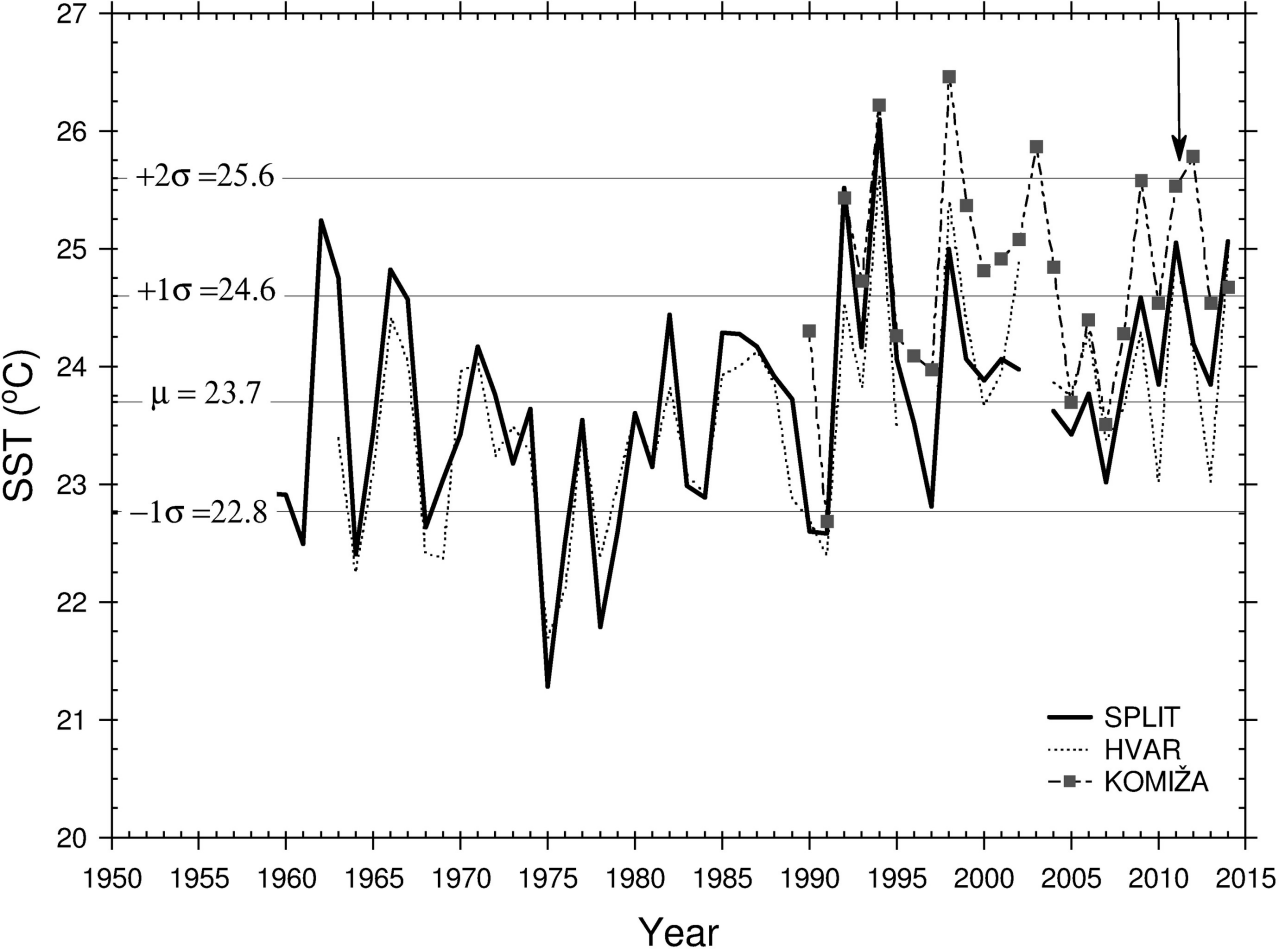
Time series of the available SST values averaged for July and August in the period between 1950 and 2015 at the coastal sea surface temperature stations Split, Hvar and Komiža.

**Fig 6:**
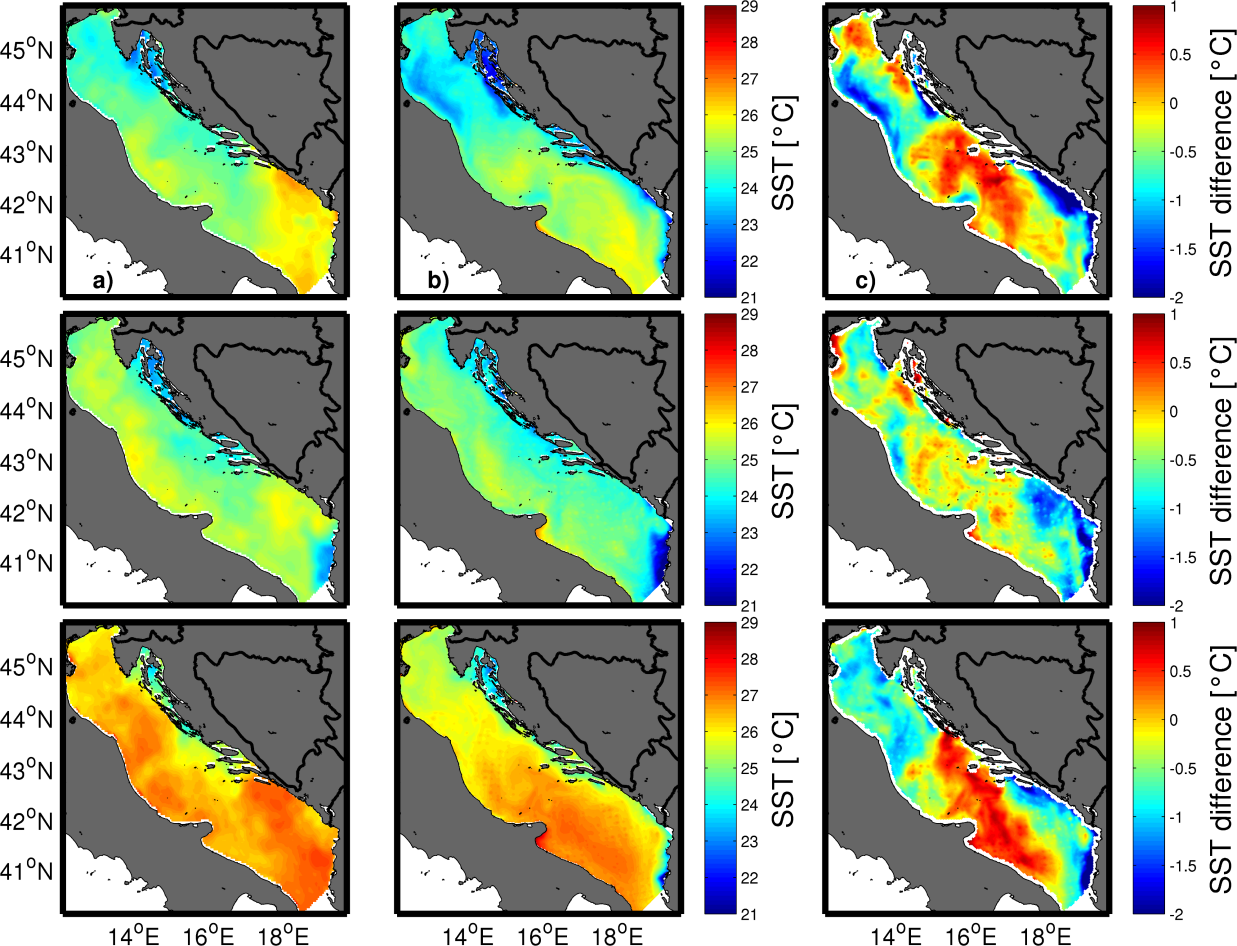
**Daily mean SST obtained from reprocessed AVHRR satellite data (column a), daily mean SST obtained from ROMS (column b) and difference between ROMS and reprocessed AVHRR SST (column c)**. The first row shows data for 23 July, second row for 14 August and third row for 1 September 2011.

**Fig 7:**
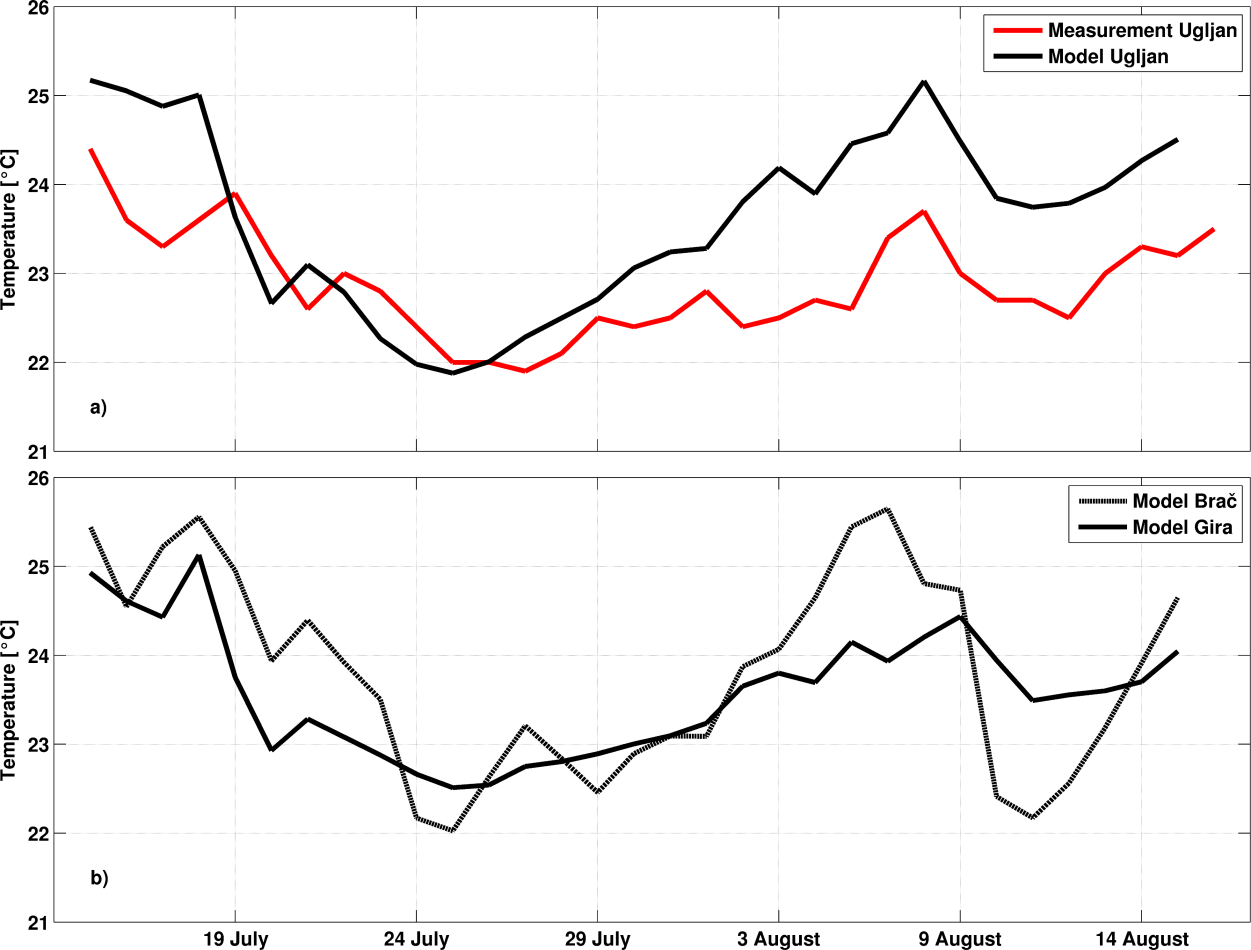
Modelled and measured time series of seawater temperature at 1 m depth within the fish farm cage near the island of Ugljan (a), and modelled time series of seawater temperature at 1 m depth within the fish farm cages near the islands Gira and Brač (b).

Detailed analysis of prevailing meteorological and oceanographic conditions was performed using all available data. During the study period weather was under the well-developed Azores high and Karachi depression [29], which over the Adriatic produce warm and almost dry weather with characteristic a well-developed sea-land breeze system. Daily air temperature means at 2 m height from the ALADIN model never dropped below 24°C on the open sea during the entire study period (Fig 4). Daily wind vectors at 10 m height blew from varying directions, though the most frequent were the northwestern and western winds (Fig 4). SST at Split and Hvar coastal stations in July and August 2011 was over one standard deviation above normal and at Komiža SST was two standard deviations above normal (Fig 5, available at http://www.izor.hr/web/guest/virtual-laboratory; [30]). The most prominent difference between ROMS and the satellite SST was seen in the open parts of the middle and southern Adriatic, though discrepancies were within values that could not impact the lower threshold of spawning temperature (Fig 6). Negative model discrepancies in front of the Adriatic coast indicate that we need to improve river implementations in the model. River discharges are determined from scaled climatological values and their temperature effect is neglected at the moment. More realistic river implementation is expected to improve modelled temperature fields. Strong temporal changes of surface temperature discrepancies in Fig 6C indicate possible effect of synoptic conditions [29], during which impact of different processes dominates. This issue needs further analysis which is beyond the scope of our paper. Anyway, we believe that for simulations we performed with Ichthyop model, mostly with passive particles, obtained discrepancy maxima of 2°C has no significant impact for the final results. Particularly because areas with maximum discrepancy are out of the assumed tuna paths. Apart from satellite SST, additional evidence of favourable spawning temperature conditions was the directly measured seawater temperature at 1 m depth inside the cage near the island of Ugljan (Fig 7) (S2 Table). Differences between measured and modelled subsurface temperatures show two patterns: before 29 July with similar values and after 29 July with similar trends but with higher discrepancies in the values. Obtained behaviour can be related to different synoptic conditions before and after 29 July. From 14 to 18 July and from 30 July to 15 August the weather was stable, warm and dry, while from 19 to 29 July atmospheric instabilities occasionally passed over the Adriatic [29]. Obtained modelled values were equal or over the measured values and during stable conditions model obviously heats too much. Although maximum discrepancies of 1.5°C are satisfactory for the simulations with passive particles, this issue requires further analysis. Moreover, we expect that higher horizontal resolution in the ROMS model simulations would increase model accuracy. Measured point is very close to the coast and it is hard to expect that 2 km resolution model is capable to reproduce precisely temperature close to such irregular coastline with many island. Higher horizontal resolution will be used in the future simulations in which we are planning to include growth and mortality temperature dependences. Anyway, with noted correlation between the model and measurements for cage location near the island of Ugljan and from simulated temperatures near the islands of Brač and Gira, it could also be assumed that spawning may have also occurred at these islands. A seawater temperature of 19.5°C is the lower threshold for wild tuna spawning in open seas of the West Mediterranean [9]. A sharp and sudden rise in temperature to 25°C recorded during spawning in captivity could be an additional favourable condition [31]. Similar behaviour was also observed in the present study (Fig 7) [11]. Modelled surface currents and currents at 10 and 30 m were in the opposite direction to the northwesterly East Adriatic Coastal Current, which is a part of the Adriatic basin-wide cyclonic circulation [32]. A similar change in summer flow direction along the eastern Adriatic coast was previously reported from direct current measurements [33] and numerical model results [34]. Modelled temperature remained above 19.5°C in the top 10 m along the presumed pathway of small tunas on the open-sea side of the Croatian islands.

### Distributions of particles in backward and forward IBM simulations

**Fig 8:**
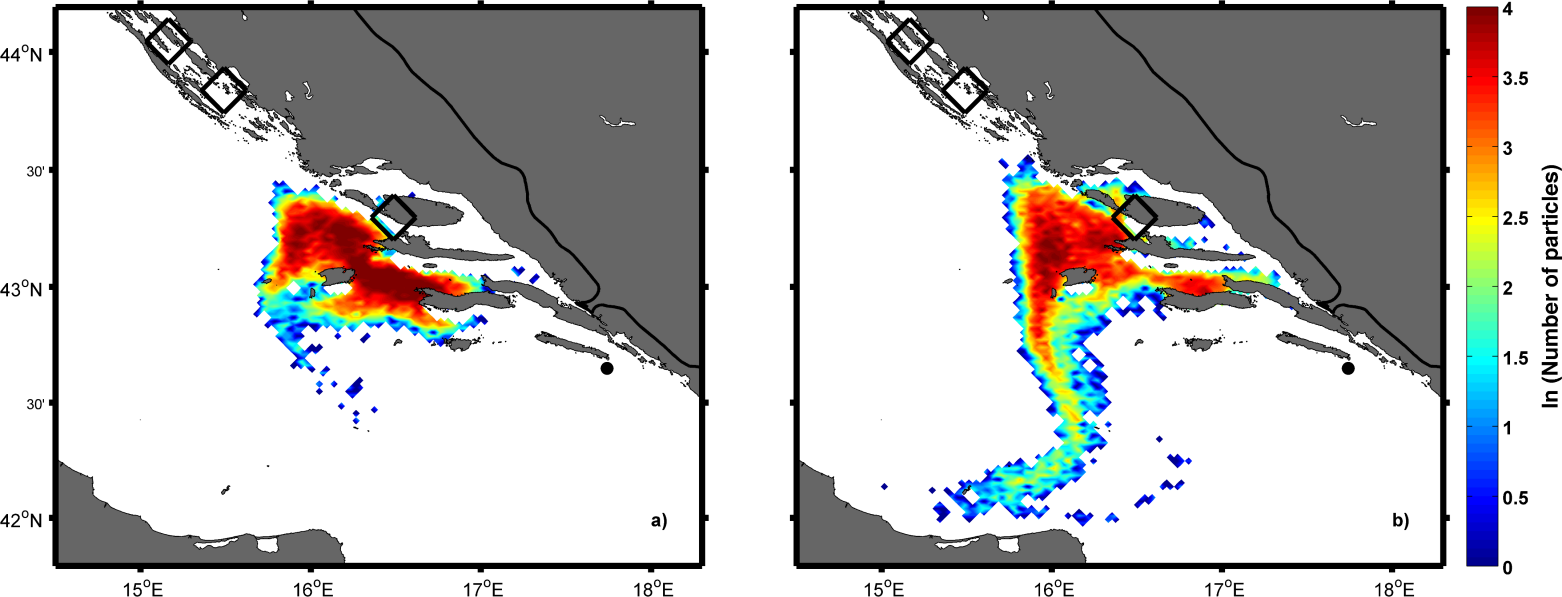
**Spatial distributions of particles obtained in the backward Ichthyop experiment with 20000 passive tracers on 5 August (a) and 19 July (b).** Origin of particles is represented by a black dot. The area of success is shown by black rectangles near the three fish farm cages.

**Fig 9:**
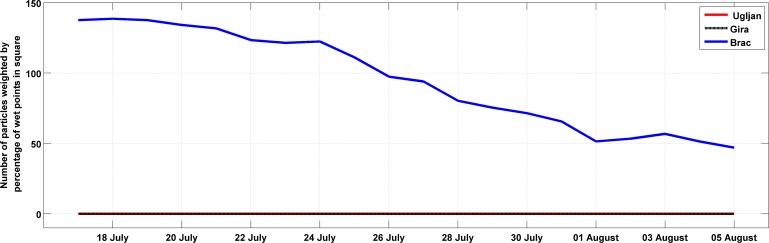
Time series of the particle arrivals within the rectangle of success around the Ugljan (red line), Gira (black) and Brač (blue) tuna farms. Particle numbers are weighted by the percentage of wet points inside the rectangles of success for the backward experiment with 20000 passive tracers.

In the backward simulations only passive “Lagrangian” experiments were performed in order to assess whether particles could successfully reach the areas around the three studied fish farms. Spatial distributions for Experiment 1 on 5 August (Fig 8A) and 19 July (Fig 8B) indicated that particles after 28 and 46 days arrived near the location of the fish cages off the island of Brač only. Spatial distributions are shown for 0300 UTC to match the daily end of the tuna spawning period and were calculated by counting the number of particles within the 2.5x2.5 km ROMS grid cell. The area of success for backward simulations was defined as a square of 225 km^2^ area where the fish cage was centrally situated. Due to the uneven distribution of land and sea at these locations, there was a need to estimate a weighting factor to obtain more reliable results for success at all three locations. Therefore, the percentage of sea points within the square with the tuna farm was multiplied by the number of particles within the square. The time series for Experiment 1 of particle arrivals near Brač (Fig 9) indicated that the number of particles increases by moving back in time with the first date of spawning. Experiments in which particles were released deeper (Experiments 2, 3 and 4) yielded similar results as Experiment 1. Reason for this is prevailing southeasterly current in the first 50 m of depth resulting in northwestward transport in the backward simulations (S1 Appendix).

**Fig 10:**
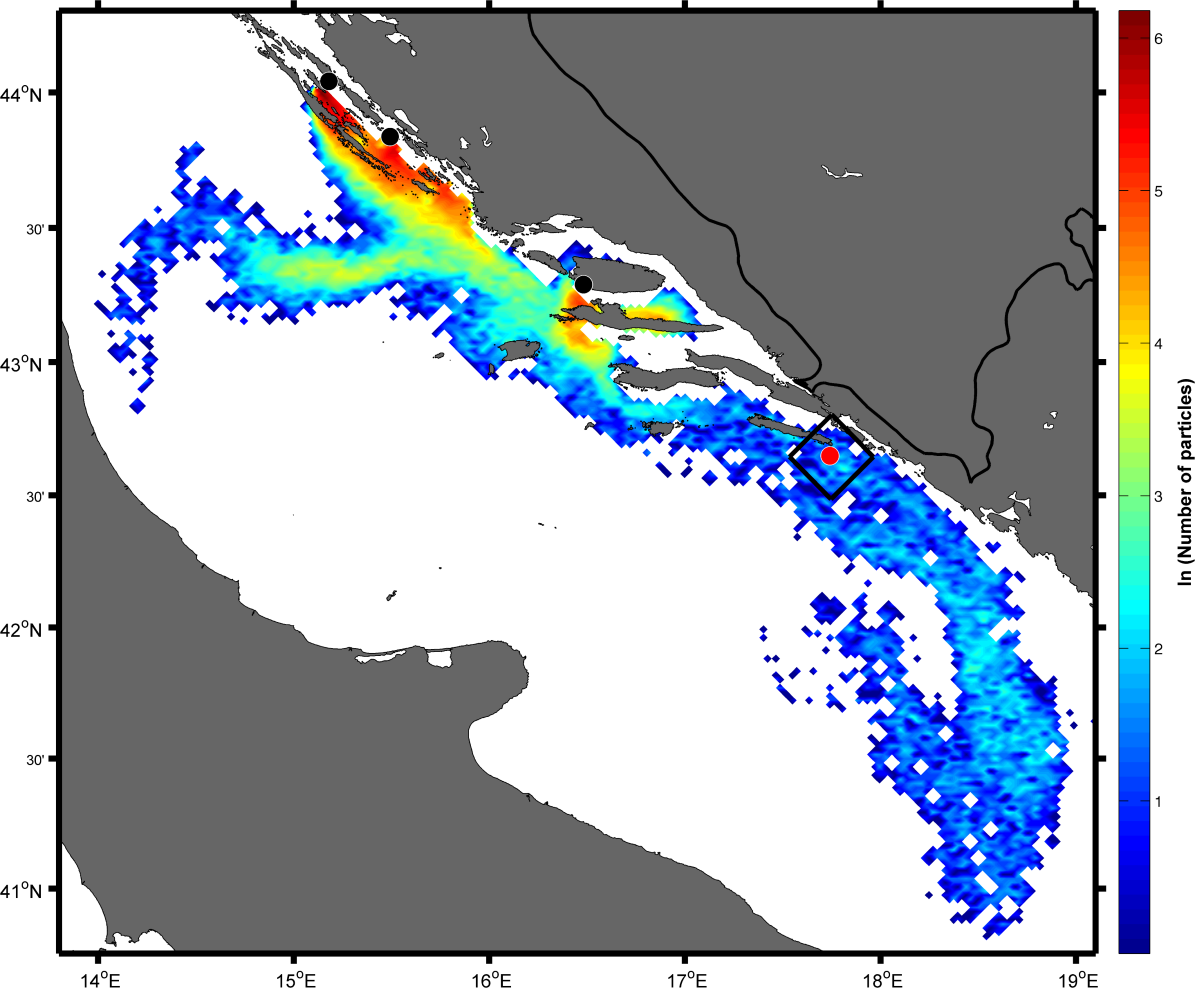
Spatial particle distribution obtained in the forward Ichthyop experiment with 20000 passive tracers from each source located near fish farms (black dots) on 1 September. The black rectangle represents the area of success around the location of the ABFT catch (red dot).

**Fig 11:**
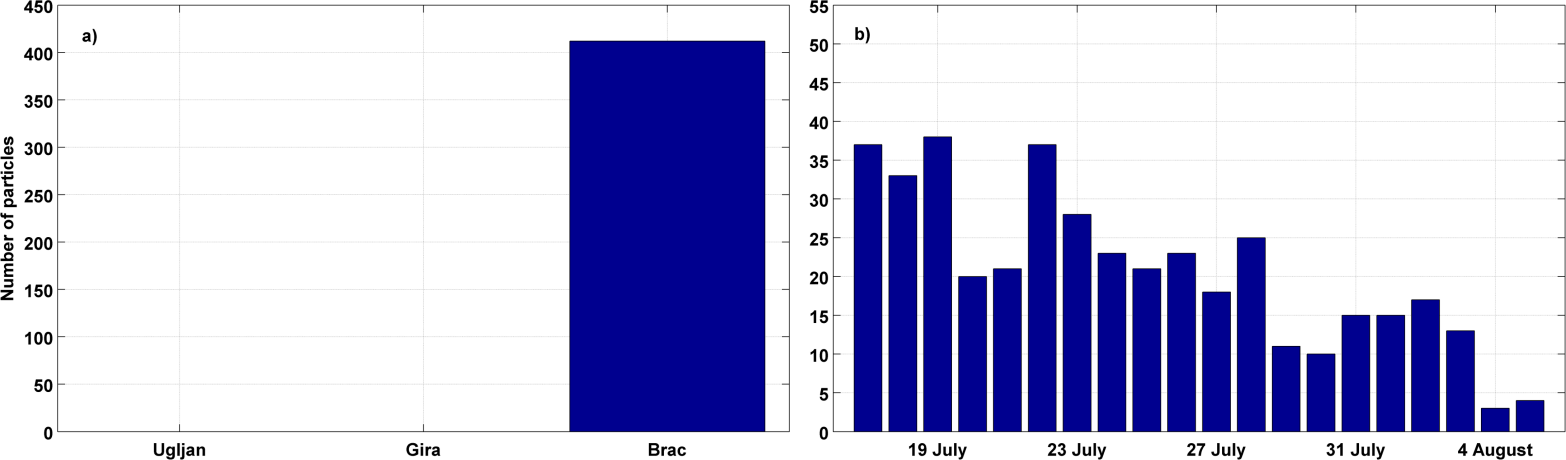
**Total particle numbers within the rectangle of success obtained in the forward Ichthyop experiment with 20000 passive tracers from each source located near the fish farm on 1 September depending on the point of origin (a).** Distribution regarding the age of particles within the rectangle of success obtained in the same experiment for the point of origin Brač (b).

In the forward simulations, the area of success was defined as a square of 625 km^2^ area where the sampling site was located centrally. The main reason for the larger area of success in the forward simulations than in backward simulations was the intention to catch more particles, as the estimated age of captured tuna suggested that they developed the ability to swim. Particle distribution on 1 September (Fig 10) clearly showed that some of particles arrived in the square near the island of Mljet. The spatial distribution on 1 September was quantified by calculating the number of particles in the area of success in respect of the point of origin and the result (Fig 11A) was the same as in the backward experiment, confirming that they originated only from Brač. Dates of their release from cages encompassed the entire assumed spawning period (Fig 11B).

**Fig 12:**
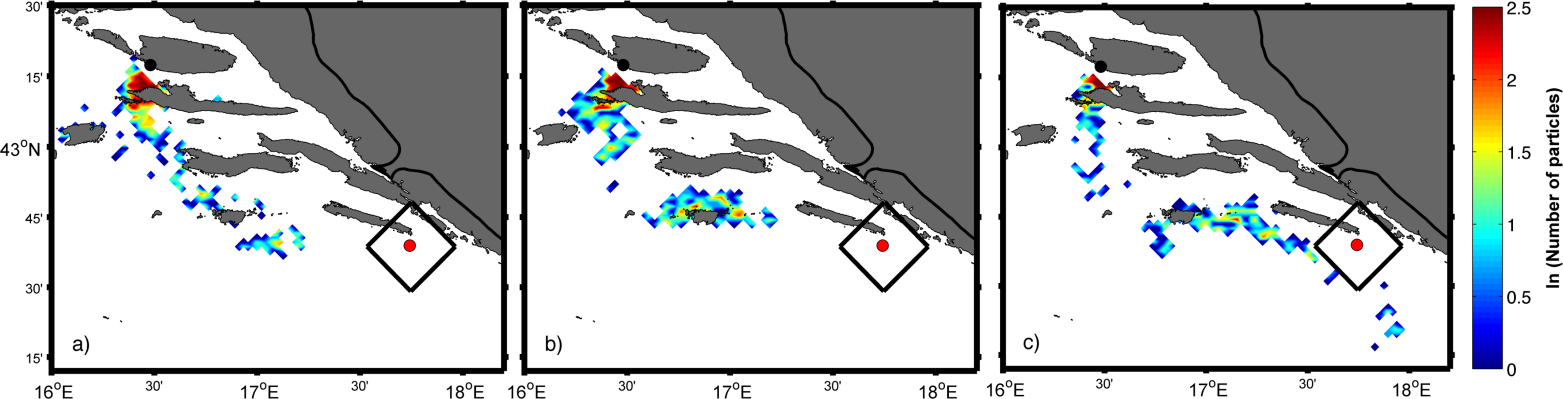
Spatial particle distribution on 20^th^ day after release obtained in the same experiment as in Fig 10 but only for the source located near the fish farm Brač (black dot). Day of snapshot: 8 August (a), 14 August (b) and 16 August (c). Black rectangle represents the area of success around the location of the ABFT catch (red dot).

**Fig 13:**
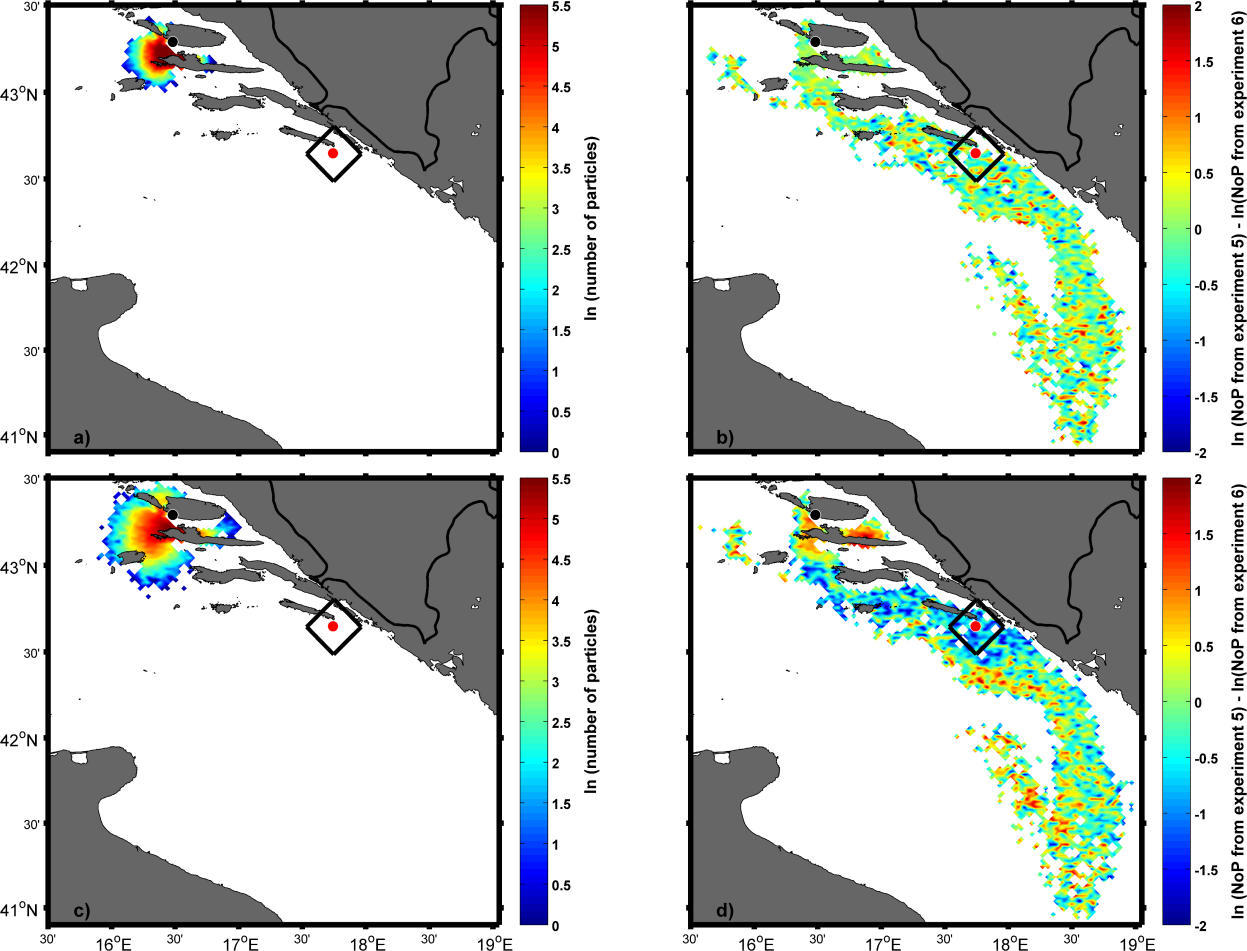
**Spatial particle distribution obtained in the forward Ichthyop experiment with 20000 particles from the source near the island of Brač on 1 September resulted only from swimming ability of particles with swimming speed of one body length/second (a) and swimming speed of four body length/second (c).** The difference on 1 September between distributions obtained in the forward Ichthyop experiment with 20000 passive tracers from the source located near the fish farm Brač (black dots) with advection and diffusion only, and distribution with the same point of origin and same number of particles with advection, diffusion and swimming speed of one body length/second (b) and of four body length/second (d).

To assess the effect of active swimming four additional forward, experiments were performed (Table 1; Experiments 6, 7, 8 and 9). Particle distribution on the 20^th^ day after release (Fig 12) showed that particles from Brač could be transported near the catch location even as the passive tracers (Table 1, Experiment 5). Active swimming was first tested as the only means of moving particles and the result showed that the distribution of particles encompassed a radius of 50 km for swimming speed of one body length/second (Fig 13A) and radius of 70 km for swimming speed of four body length/second (Fig 13C) Combination of both swimming options and passive movements (Experiments 6 and 7) yielded that particle distributions for both used swimming speeds (Fig 13B and 13D) were similar to particle distributions with passive displacement only (Experiment 5). The reason for such small deviation from the distribution based on passive movement is mainly the fact that the direction in which particles are swimming is randomly chosen in each time step.

To test the possibility of particles arriving from the Ionian Sea at the sampling site, in Experiment 10 particles were released from the southern edge of the domain (Fig 1). At the end of simulation, all particles were transported outside the model domain towards the Ionian Sea due to the prevailing southeasterly surface flow.

## Discussion

In a fluctuating ecosystem, such as the sea, sustainability of fish population is strongly determined by successful reproduction. Hence, knowledge on fish reproduction and its ecologically important habitats, such as spawning and nursery grounds, is required to allow scientific advisors and regulators to better manage their stocks.

An incidental finding of scombrids in September 2011 in the southern Adriatic raises the issue of the possible tuna spawning in the Adriatic Sea. In addition to the scientific significance, strict legislative regulation of tuna stock concerning farming and fisheries, is additional motivation to resolve this dilemma.

The main goal of this study was to discover the origin of small tuna fish caught in the southern Adriatic. Therefore, a multidisciplinary investigation was undertaken encompassing purse seine monitoring data, their genetic and phenotypic analysis, analysis of the prevailing meteorological and oceanographic conditions and finally the numerical study of the early tuna stage dynamics using coupled modelling system ROMS—Ichthyop. Similar approach with genetic analysis and circulation modelling used to investigate pan-Atlantic connectivity of endangered green turtles [35] is another example of multidisciplinary investigation widely used nowadays in revealing population structures and their spatial distributions. Although genetic and phenotypic analyses were performed for all collected specimens, further numerical experiments and discussions were focused on bluefin tuna.

**Fig 14:**
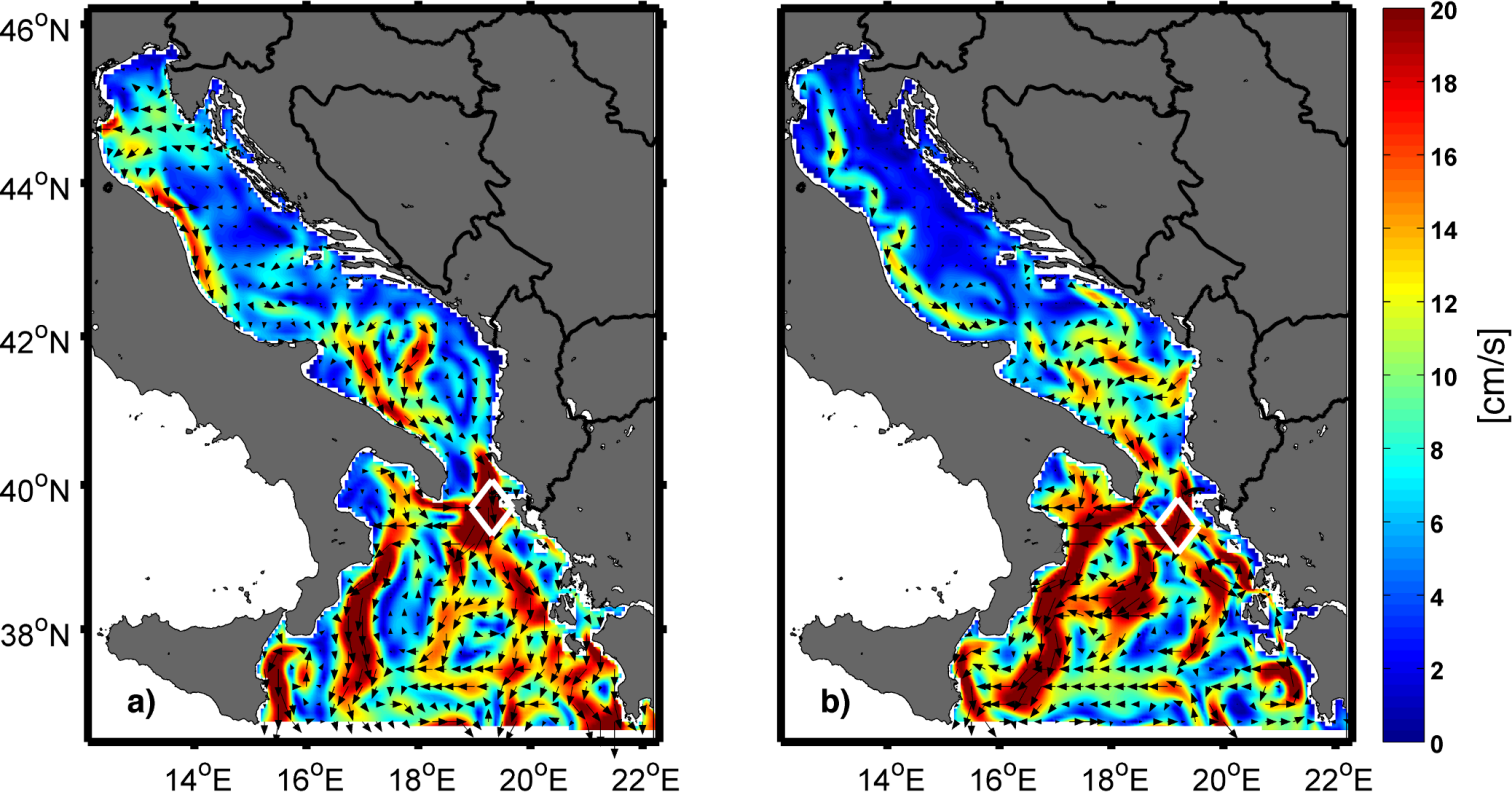
**Daily mean sea currents calculated by the Mediterranean model at 1m depth on 23 July (a) and 19 August (b).** Vectors are plotted at every fourth grid point. A white rectangle denotes the area with maximum speed during the selected date.

Phenotypic analyses (length-length relationship and positive allometric growth) were in line with previously published data for each analysed scombrid species [23] and the obtained age for all collected specimens (between 30–40 days) suggested their possible spawning in the Adriatic. There are several possibilities for the spawning location of the collected small tunas. The closest known area of natural tuna spawning to the sampling site is the eastern Sicilian coast [12, 13]. An average surface current of 20 cm/s obtained during August 2011 by the Mediterranean model (Fig 14) along the possible tuna pathway and ability of tuna to swim 20 days after spawning with speeds ranging from 8 cm/s on 20^th^ to 15 cm/s on 48^th^ day would allow for crossing about 700 km within 35 days from the eastern Sicilian coast to the sampling site. Multiplying swimming speeds with factors ranging from 2 to 4 would allow further easier and faster arrival from the eastern Sicilian coast to the island Mljet. However, the prevailing southerly direction of the surface flow and its complex structure most likely create an unsurpassable obstacle for small tunas to reach the southern coast of the island Mljet. An additional argument to assess the eastern Sicilian coast as an unlike spawning location is the agreement of the southerly surface flow from the Mediterranean model with the ROMS model results used by Ichthyop. Moreover, prevailing southerly surface flow transported all particles in Ichthyop simulation originating from the Strait of Otranto towards the Ionian Sea instead towards sampling site. Since natural tuna spawning in the Adriatic is questionable, the working hypothesis assumed that the discovered small tunas spawned in one of the farms positioned along the eastern Adriatic coast north of the sampling site. Upon spawning, they were carried southwards by the prevailing SE current for about 20 days. After approximately 20 days, the tuna could actively swim independent of the hydrodynamic environment [36], allowing them to arrive at the sampling place within their estimated age.

An important part of the study was the numerical modelling system, which enabled the testing of the hypothesis on the spawning location. A similar modelling setup with a hydrodynamic and individual based model (IBM) has been extensively used for the early stages of many species (sardines, anchovy), though published data and modelling of early stage dynamics of tuna are scarce. According to our knowledge, this study is the first attempt to numerically model early stage dynamics of tuna in the Adriatic Sea. Using hourly ROMS output and horizontal resolution of 2.5 km we preserved all important physical processes which occur in the Adriatic [37]. Anyway, taking into account complex topography further improvements could be made for the simulations along the eastern Adriatic coast by applying finer spatial resolution and nesting procedures [34]. Due to the lack of information and knowledge of the processes during the early life of tuna, numerical simulations were conducted with the assumption that particles in the Ichthyop model were passive. Introduction of additional processes and dependences of the tuna eggs and larvae in the simulations, such as buoyancy, diel vertical migrations, temperature dependences, would likely increase the model accuracy and reliability of the results. Some processes are already included in the Ichthyop simulations, though most of the parameterizations required have been obtained for areas outside the Adriatic. The presently introduction of the Ichthyop default diel vertical movement [21] resulted in a pile up along the western Adriatic coast during the forward experiment. Therefore, in order to proceed with similar numerical investigations, better parameterizations from the laboratory and field experiments in the Adriatic are needed. ARGO drifter data (S1 Appendix) were used in our study for verification of the ROMS reproduced circulation. Unfortunately it was not possible to use the drifter data for assessing the connectivity between supposed spawning location and sampling site and comparison between passive and active movements since ARGO drifter was moving in the Jabuka Pit far away from the presumed tuna pathway [38].

Furthermore, the present study clearly showed the importance of accurately simulated physical fields, particularly the current field responsible for the early tuna stage transport. Realistic numerical simulations revealed a current reversal along the eastern Adriatic coast, from a NW to SE flow. Occurrence of the current reversal has previously been observed [33] and modelled [34] in this area, particularly during summer. The impact of reversal has never been studied with dispersion simulations to reveal the area connectivity and its impact on marine ecology. Connectivity studies in the Adriatic Sea were more focused on decadal time scales and basin as a whole [39, 40]. Without the reversed SE flow, tuna eggs and larvae would not be able to travel south to the sampling site. With a NW flow, small tuna would move northwards of the fish farms in the middle Adriatic and it is questionable whether they would survive in the northern coastal area of the Adriatic. Reproduction of the temperature field is also very important as the actual onset of spawning behaviour is temperature related [9].

Results of the numerical simulations with the Ichthyop model, both backward and forward, indicate that the commercial tuna cages in the middle Adriatic coastal area are a possible spawning location, while the tuna farm off the coast of the island of Brač was the most probable origin of the collected specimens. Due to the presence of the middle Adriatic gyre, passive particles in the backward experiments could not reach the other tuna farms at Gira and Ugljan, thus eliminating them as a possible origin, despite records of natural spawning in these cages [11]. The forward experiment indicated that the particles from the Brač tuna farm could arrive 20 to 50 km away from the sampling site within 20 days even as passive tracers no matter of releasing date. This result indicates that prevailing current direction is the most important factor for arrival of small tunas at the sampling site and sensitivity on time and date of release is not precedent [41]. We are aware that passive displacement by currents only and with addition of swimming with randomly chosen direction was not the best choice, but in the lack of better model swimming parameterizations and knowledge of tuna swimming, it represented a usable tool to study small tuna displacement. Even if this displacement was the slowest one and in most cases excluded maximal displacement possible that could arrive from matching the directions of swimming and prevailing currents. By adopting the hypothesis that the Brač tuna farm is the spawning location, we have valuable material for future testing of numerical model improvements. It is very rare to have exact location of spawning. Typically, this is a larger area with a high possibility of spawning, whereas in this case, we have virtually a point source in which spawning occurred (fish farm cage). A well-defined spawning location gives high confidence in assessing both model results, hydrodynamic ROMS and IBM Ichthyop.

## Conclusions and future work

Finally, it can be concluded that based on the performed simulations, the most probable spawning location for the Atlantic bluefin tuna found in the Adriatic was the commercial tuna cages off the southern coast of Brač Island. This is possible, as natural spawning has previously been reported within commercial tuna farming facilities in the Adriatic [11]. However, the fact that the two other species most likely opportunistically use the positive environmental conditions and spawn in the same area could led us to further investigate the possibility that bluefin tuna could do the same.

This multidisciplinary study, supported by genetic and phenotypic analysis, is the first attempt to numerically model early tuna stage dynamics in the Adriatic Sea. There are few similar numerical investigations for other areas, and therefore it was impossible to include more biology of the tuna early stage without increasing the uncertainty in the model solutions. Numerical experiments were performed with passive particles, although modelled specimens do not fall entirely into passive swimmers. The effects of active swimming were tested in the forward experiments. Active swimming implemented with passive movements, with the assumption of randomly chosen swimming direction in each time step, had no significant influence on the IBM results. Additional testing should be made for swimming patterns parallel or perpendicular to the prevailing current orientation [42].

Many improvements are possible for the hydrodynamic and IBM models, and by adopting the tuna farm off the southern Brač coast as the spawning location, we have valuable material to assess both model enhancements. Higher horizontal resolution of the ROMS model would allow for more detailed reproduction of circulation in a complex topographic area such as the eastern Adriatic, with many structures like vortices, jets and wakes that may be important for the advection of ichthyoplankton. More realistic introduction of swimming, diel vertical migrations, and temperature related processes such as growth and mortality, and impact of food availability in the IBM simulations would require new parameterizations based on the laboratory and field experiments for the Adriatic.

## Supporting information

S1 Table Age and length of measured fish. (XLSX)

S2 Table Time series of seawater temperature measured at 1 m depth in the fish farm cage near Ugljan. (TXT)

S1 Appendix Description of the hydrodynamic model and verification of its results. (DOCX)

S1 Fig Age frequency distribution of Atlantic bluefin tuna, *Thunnus thynnus* (Linnaeus, 1758), bullet tuna, *Auxis rochei* (Risso, 1810) and little tunny, *Euthynnus alletteratus*, Rafinesque, 1810 collected with commercial purse seiners operating in the southeastern Adriatic, September 2011. (TIF)

## References

[1] Collette BB. Mackerels, molecules and morphology. In: Séret B, Sire JY, editors. Proceedings 5th Indo-Pacific Fish Conference; 1997 Nov 3–5; Nouméa. Paris Soc Fr Ichtyol; 1999. pp. 149–164.

[2] Block BA, Teo SLH, Walli A, Boustany A, Stokesbury MJW, Farwell CJ, et al Electronic tagging and population structure of Atlantic bluefin tuna. Nat. 2005; 434: 1121–1127. doi: 10.1038/nature03463 10.1038/nature03463 15858572

[3] Collette BB, Carpenter KE, Polidoro BA, Juan-Jordá MJ, Boustany A, Die DJ, et al High value and long life—Double jeopardy for tunas and billfishes. Science. 2011; 333: 291–292. doi: 10.1126/science.1208730 21737699 10.1126/science.1208730

[4] Juan-Jordá MJ, Mosqueira I, Cooper AB, Dulvy NK. Global population trajectories of tunas and their relatives. Proc Nat Acad Sci. 2011; 51: 20650–20655. doi: 10.1073/pnas.1107743108 10.1073/pnas.1107743108 PMC3251139 22143785

[5] Benetti DD, Partridge GJ, Buentello A. Advances in tuna aquaculture. Oxford: Elsevier; 2016.

[6] Tičina V, Katavić I, Grubišić L. Growth indices of small northern bluefin tuna (*Thunnus thynnus*, L.) in growth-out rearing cages. Aquaculture. 2007; 269: 538–543. doi: 10.1016/j.aquaculture.2007.05.029

[7] De la Gándara F, Ortega A, Buentello A. Chapter 6—Tuna Aquaculture in Europe. In: Advances in Tuna Aquaculture, San Diego: Academic Press; 2016 pp. 115–157. doi: 10.1016/B978-0-12-411459-3.00005–9

[8] Karakulak S, Oray I, Corriero A, Deflorio M, Santamaria N, Desantis S, et al Evidence of a spawning area for the bluefin tuna (*Thunnus thynnus L*.) in the Eastern Mediterranean. J. Appl. Ichthyol. 2004; 20:318–320. doi: 10.1111/j.1439-0426.2004.00561.x

[9] Gordoa A, Carreras G. Determination of Temporal Spawning Patterns and Hatching Time in Response to Temperature of Atlantic Bluefin Tuna (*Thunnus thynnus*) in the Western Mediterranean. PLoS ONE. 2014; 9(3): e90691 doi: 10.1371/journal.pone.0090691 24608107 10.1371/journal.pone.0090691 PMC3946554

[10] De la Gándara F, Ortega A, Belmonte A, Mylonas CC. Spontaneous spawning of Atlantic bluefin tuna Thunnus thynnus kept in captivity. In: Proceedings of the Aquaculture Europe 2011. European Aquaculture Society; 2011 Oct 18–21; Rhodes, Greece. 2011. pp. 249–250. doi: 10.13140/2.1.2976.7682

[11] Grubišić L, Šegvić-Bubić T, Lepen Pleić I, Mišlov K, Tičina V, Katavić I, et al Morphological and genetic identification of spontaneously spawned larvae of captive Bluefin Tuna *Thunnus thynnus* in the Adriatic Sea. Fish. 2013; 38(9): 410–417. doi: 10.1080/03632415.2013.839439

[12] Corriero A, Karakulak S, Santamaria N, Deflorio M, Spedicato D, Addis P, et al Size and age at sexual maturity of female bluefin tuna (*Thunnus thynnus L*. *1758*) from the Mediterranean Sea. 2005; J. Appl. Ichthyol. 21: 483–486. doi: 10.1111/j.1439-0426.2005.00700.x

[13] Druon J-N, Fromentin J-M, Hanke AR, Arrizabalaga H, Damalas D. Habitat suitability of the Atlantic bluefin tuna by size class: An ecological niche approach. Prog Oceanogr. 2016; 142: 30–46.

[14] Alemany F, Quintanillab L, Velez-Belchíc P, Garcíab A, Cortésb D, Rodríguezd JM, et al Characterization of the spawning habitat of Atlantic Bluefin tuna and related species in the Balearic Sea (western Mediterranean), Prog Oceanogr. 2010; 86 (1–2): 21–38. doi: 10.1016/j.pocean.2010.04.014

[15] Uchida RN. Synopsis of biological data on Frigate Tuna *Auxis thazard* and Bullet Tuna *A*. *rochei*. FAO Fish Synops. 1981; 124: 63 p.

[16] Alemany F. Ictioplancton del Mar Balear [dissertation]. Palma de Mallorca: University of Illes Balears; 1997.

[17] Kahraman AE, Alicli TZ, Akayli T, Oray K. Reproductive biology of little tunny, *Euthynnus alletteratus* (Rafinesque), from the north-eastern Mediterranean Sea, J Appl Ichthyol. 2008; 24(5): 551–554. doi: 10.1111/j.1439-0426.2008.01068.x

[18] Cermeño P, Quílez-Badia G, Ospina-Alvarez A, Sainz-Trápaga S, Boustany AM, Seitz AC, et al Electronic tagging of Atlantic bluefin tuna (*Thunnus thynnus*, L.) reveals habitat use and behaviors in the Mediterranean Sea. PLoS ONE. 2015; 10: e0116638 doi: 10.1371/journal.pone.0116638 25671316 10.1371/journal.pone.0116638 PMC4324982

[19] Shchepetkin AF, McWilliams JC. A method for computing horizontal pressure-gradient force in an oceanic model with a non-aligned vertical coordinate. J Geophys Res. 2003: 108(C3). doi: 10.1029/2001JC001047

[20] Shchepetkin AF, McWilliams JC. The Regional Ocean Modeling System (ROMS): A split-explicit, free-surface, topography following coordinates ocean model. Ocean Modell. 2005; 9(4): 347–404. doi: 10.1016/j.ocemod.2004.08.002

[21] Lett C, Verley P, Mullon C, Parada C, Brochier T, Penven P, et al A Lagrangian tool for modelling ichthyoplankton dynamics. Environ Model Softw. 2008; 28: 1210–1214. doi: 10.1016/j.envsoft.2008.02.005

[22] Librado P, Rozas J. DnaSP v5: a software for comprehensive analysis of DNA polymorphism data. Bioinformatics. 2009; 25: 1451–1452. doi: 10.1093/bioinformatics/btp187 19346325 10.1093/bioinformatics/btp187

[23] La Mesa M, Sinopoli M, Andaloro F. Age and growth of juvenile Bluefin tuna (*Thunnus thynnus*) from the Mediterranean Sea (Sicily, Italy). Sci 3 2005; 69(2):241–249.

[24] Santamaria N, Acone F, Deflorio M, Potoschi A, Gentile R, Megalofonou P, et al Età ed accrescimento in giovani di Tombarello (*Auxis rochei* Risso, 1810) nei mari meridionali italiani. Biol Mar Medit. 2000; 8(1): 765–770.

[25] Santamaria N, Deflorio M, De Metrio G. Preliminary study on age and growth of juveniles of *Sarda sarda*, Bloch and *Euthynnus alletteratus*, Rafinesque, caught by clupeoids purse seine in the Southern Italian Seas. Collect Vol Sci Pap ICCAT. 2005; 56(2): 630–643.

[26] Brickman D, Smith PC. Langrangian stochastic modelling in coastal oceanography. J Atmos Ocean Tech. 2002; 19:83–99.

[27] Mariani P, MacKenzie BR, Iudic D, Bozec A. Modelling retention and dispersion mechanisms of bluefin tuna eggs and larvae in the northwest Mediterranean Sea. Progr Oceanogr. 2010; 86: 45–58. doi: 10.1016/j. pocean.2010.04.027

[28] Reglero P, Zaragozna N, Blanco E, de la Gandara F, Torrers AP, Ortega A. Routine swimming speed of bluefin tuna larvae measured in the laboratory. 39th Annual Larval Fish Conference; 2015 Jul 12–17; Vienna, Austria.

[29] DHMZ (Meteorological and Hydrological Service). Reviews NO23: Climate Monitoring and Assessment for 2011. DHMZ; Oct 2013.

[30] Grbec B, Bajić A, ViLab team. Virtual laboratory [cited 2016 Jul 21]. Institute of Oceanography and Fisheries Split. Meteorological and Hydrological Service, Zagreb. Available from: http://www.izor.hr/web/guest/virtual-laboratory

[31] Mylonas CC, Bridges C, Gordin H, Belmonte Rios A, Garcia A, De la Gandara F. Preparation and administration of gonadotropin-releasing hormone agonist (GnRHa) implants for the artificial control of reproductive maturation in captive-reared Atlantic bluefin tuna (*Thunnus thynnus thynnus*). Rev Fish Sci 2002; 15: 183–210. doi: 10.1080/10641260701484572

[32] Cushman-Roisin B, Poulain PM. Circulation In: Cushman-Roisin B, Gačić M, Pulain PM, Artegiani A, editors. Physical Oceanography of the Adriatic Sea. Dordrecht: Kluwer Academic Publishers; 2001 pp. 67–109.

[33] Zore-Armanda M. The system of currents in the Adriatic Sea. Études et revues–Conseil général des peches pour la Méditerranée, FAO, Rome, 1968; 34: 1–48.

[34] Orlić M, Beg Paklar G, Pasarić Z, Grbec B, Pasarić M. Nested modeling of the east Adriatic coastal waters. Acta Adriat. 2006; 47(Suppl.): 219–245.

[35] Naro-Maciel E, Hart KM, Cruciata R, Putman NF. DNA and dispersal models highlight constrained connectivity in a migratory marine megavertebrate. Ecography. 2016; 40: 586–597. doi: 10.1111/ecog.02056

[36] Mather FJ, Mason JM, Jones AC. Historical Document: Life History and Fisheries of Atlantic Bluefin Tuna. NOAA Technical Memorandum, NMFS-SEFSC 1995; pp. 1–165.

[37] Putman NF, He R. Tracking the long distance dispersal of marine organisms: sensitivity to ocean model resolution. J R Soc Interface. 2013; 10(81): 20120979 doi: 10.1098/rsif.2012.0979 23349437 10.1098/rsif.2012.0979 PMC3627105

[38] Fossette S, Putman NF, Lohmann KJ, Marsh R, Hays GC. A biologist’s guide to assessing ocean currents: a review. Mar Ecol Prog Ser. 2012; 457: 285–301. doi: 10.3354/meps09581

[39] Paco M, Schiavina M, Rossetto M, Gatto M, Fraschetti S, Casagrandi R. Looking for hotspots of marine metacommunity connectivity: a methodological framework. Sci Rep. 2016; 23705 doi: 10.1038/srep23705 10.1038/srep23705 PMC4814777 27029563

[40] Bray L, Kassis D, Hall-Spencer JM. Assessing larval connectivity for marine spatial planning in the Adriatic. Mar Environ Res. 2017; 125: 73–81. https://doi.org/10.1016/j.marenvres.2017.01.006 28187325 10.1016/j.marenvres.2017.01.006

[41] Putman NF, Lumpkin R, Sacco AE, Mansfield KL. Passive drift or active swimming in marine organisms? Proc R Soc B. 2016; 283:20161689 http://dx.doi.org/10.1098/rspb.2016.1689 27974518 10.1098/rspb.2016.1689 PMC5204149

[42] North EW, Gallego A, Petitgas P. Manual of recommended practices for modelling physical–biological interactions during fish early life. ICES Cooperative Research Report No. 295. 2009; pp. 1–111.

