# Supplementary material for: Spillover of the Atlantic bluefin tuna offspring from cages in the Adriatic Sea: A multidisciplinary approach and assessment
Source: PLoS One. 2017 Nov 30;12(11):e0188956. doi: 10.1371/journal.pone.0188956 (PMC5708836; doi:10.1371/journal.pone.0188956)
Supplement: S1 Appendix — (DOCX) [file pone.0188956.s003.docx]

**S1 Appendix: Description of the hydrodynamic model and verification of its results**

The ROMS model is a finite difference, free-surface, primitive equations model with hydrostatic and Boussinesq approximations, whose physical and numerical algorithms are described in detail by Shchepetkin and McWilliams [20, 21]. In this application, the ROMS model domain covers the entire Adriatic, with a rectangular grid having horizontal resolution of 2.5 km and 22 unequally spaced s levels along the vertical. The horizontal grid consists of 320x108 points. The model bathymetry is based on 7.5 second resolution depth field derived during the DART project (Dynamics of the Adriatic in Real-Time) at the NATO Undersea Research Centre (NURC) using an inverse distance weighted interpolator (Rixen et al., 2006). Fine resolution NURC bathymetry data were bin averaged on the ROMS grid and smoothed according to the method proposed by Dutour et al. (2009). Surface momentum, heat and water fluxes were calculated using atmospheric fields from the operational ALADIN model (Tudor et al., 2013), having a horizontal resolution of 8 km for scalar fields (air pressure, air temperature, relative humidity, cloudiness, precipitation, and shortwave radiation) and 2 km resolution for wind fields. ALADIN fields with temporal resolution of 3 hours were bi-linearly interpolated into the Adriatic-scale model grid to perform interactive calculations of surface fluxes. The ROMS model, in addition to atmospheric forcing, was also forced with river inflows, tides and water mass exchange through the Strait of Otranto. Along the Adriatic coast, 41 rivers are discharging and their climatological flow rates (Raicich, 1994) were used in the simulations. Tidal forcing was applied on the open boundary taking into account seven tidal harmonics (M2, S2, N2, K2, K1, O1 and P1) crucial for the Adriatic dynamics. The open boundary conditions for the free surface, temperature, salinity, and velocity are taken from the wider Adriatic model AREG operationally run under the Adriatic Forecasting System (AFS) (Oddo et al., 2006). For the barotropic part, a modified Flather scheme was used (Flather, 1976), while for baroclinic velocity and tracers (temperature and salinity) a combination of Orlanski-type radiation boundary conditions with nudging was applied (Marchesiello et al., 2001). ROMS simulation was run for the period from 1 January 2011 to 2 September 2011. Initial temperature and salinity fields were homogeneous, while the current field started from a state of rest. The start of simulations six and a half months prior to the beginning of use of the ROMS model results in the Ichthyop model ensured the development of realistic ROMS fields and reduced influence of unrealistic initial conditions and related spurious wave generation. In order to further minimize spurious oscillations, all forcing were linearly spun up from zero to maximum values over the first day of simulation.

The quality of the ROMS model results was assessed with satellite sea surface temperatures (SST), subsurface temperature obtained at the tuna farm, drifting path of the ARGO float and its temperature and salinity profiles. Daily averages of satellite SST interpolated for the Adriatic Sea through an optimal interpolation algorithm were downloaded from the Copernicus Marine Environment Monitoring Service database (marine.copernicus.eu). SST fields were created by CNR (Consiglio Nazionale delle Richerce) for the Mediterranean Sea and have reprocessed the Pathfinder V5.2 (PFV52) AVHRR data over the period November 1981 – December 2012 to provide daily gap-free maps (L4) at the original PFV52 resolution at 0.0417° x 0.0417°. Seawater temperature at 1 m below the surface was measured inside the cage at the tuna farm located 200 m off the coast of island of Ugljan [11] and was also compared with the corresponding ROMS results. Moreover, the drifting path of the ARGO float (code 1900848, available at <http://www.coriolis.eu.org>) together with vertical profiles of temperature and salinity measured every 5 days, were used for ROMS model evaluation.

Long-lasting warm and dry weather conditions occurred in summer 2011, producing a deeper surface layer with a thermocline depth of about 35 m (Fig A), as shown on the thermohaline vertical profiles measured by the ARGO float located at Jabuka Pit during the study period. Every fifth day the ARGO float made a profiling, and two profiles are plotted in Fig A. The modelled profiles indicate that ROMS caught not only the horizontal surface temperature distribution, but it also matched realistic salinity and temperature profiles. Discrepancies in salinity profiles can be neglected due to their small values. Modelled surface currents showed prevailing southerly and southeasterly flow along the eastern Adriatic coast (Fig B) during the entire study period. Currents were stronger and less variable in the area near the island of Mljet than currents near the island of Brač, where weakening and direction change occurred.

Verification of ROMS reproduced circulation came from the calculation of the ARGO drifter path (Fig C). ROMS passive tracers were released every fifth day in time and location corresponding to ARGO drifter surfacing. The best agreement between the modelled and realistic drifter was obtained for the ROMS drifter floating at 120 m although the ARGO drifter was floating at depths between 150 and 200 m. ROMS drifters were tracked until the end of simulation on 2 September and their pathways roughly matched the pathway of the ARGO float (Fig C).


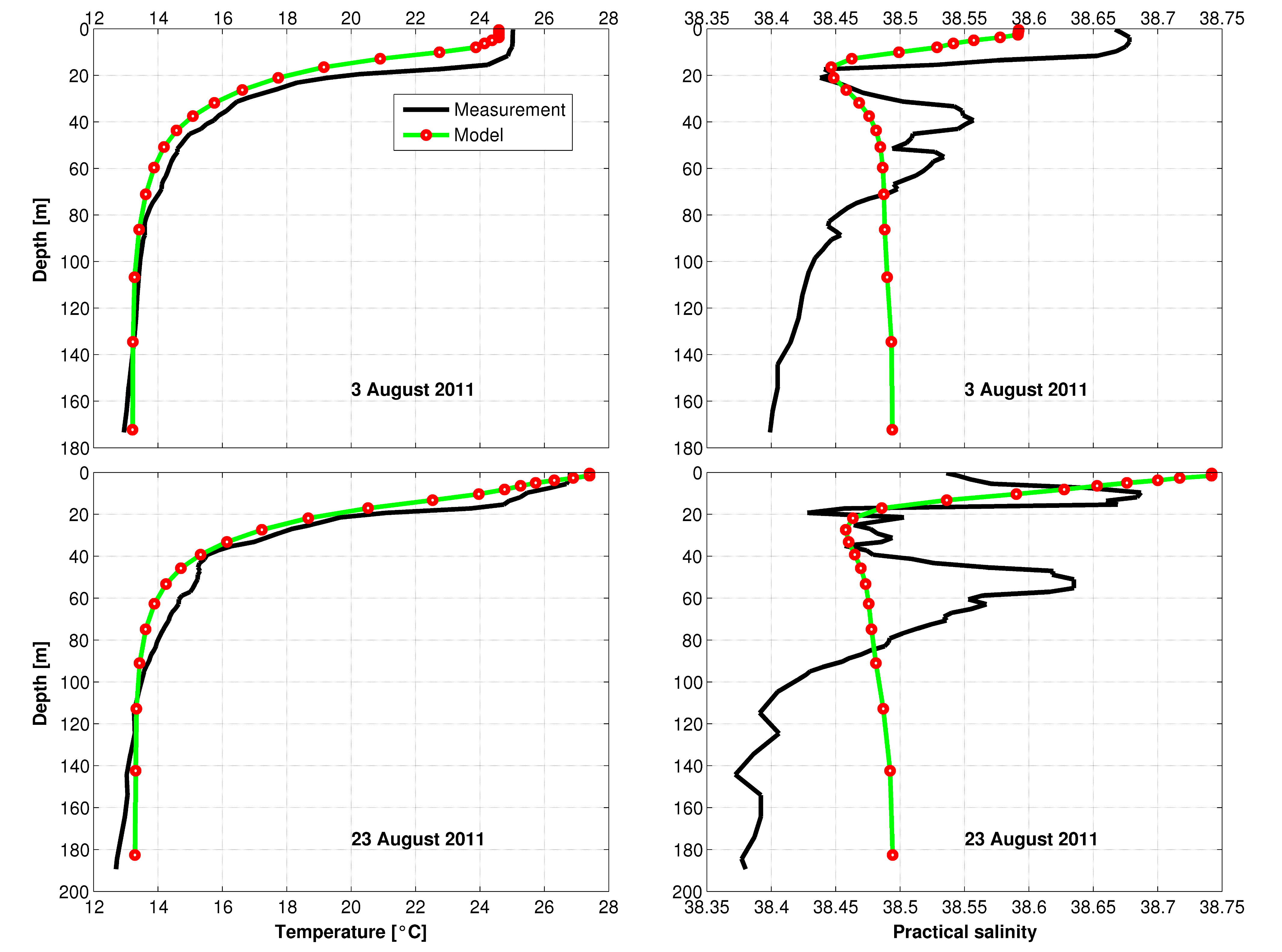


**Fig A. Modelled and measured temperature and salinity profiles on 3 and 23 August 2011.** Measured profiles were obtained by the ARGO float.

**
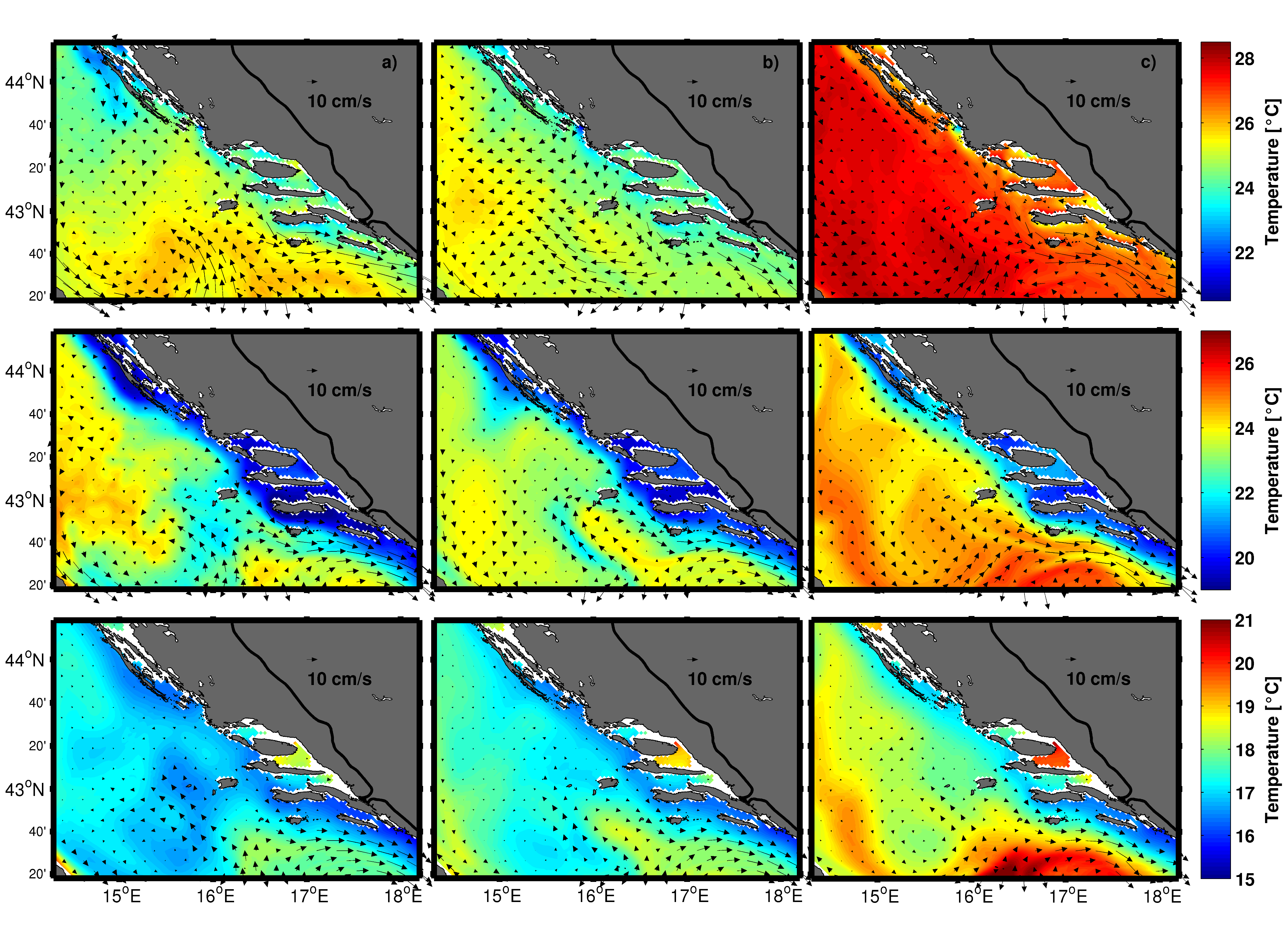
**

**Fig B. Daily mean temperatures and sea currents calculated by ROMS at depth of 1 m (first row), 10 m (second row) and 30 m (third row), on 22 July (column a), 8 August (column b) and 25 August (column c).** Vectors are plotted at every fourth grid point.


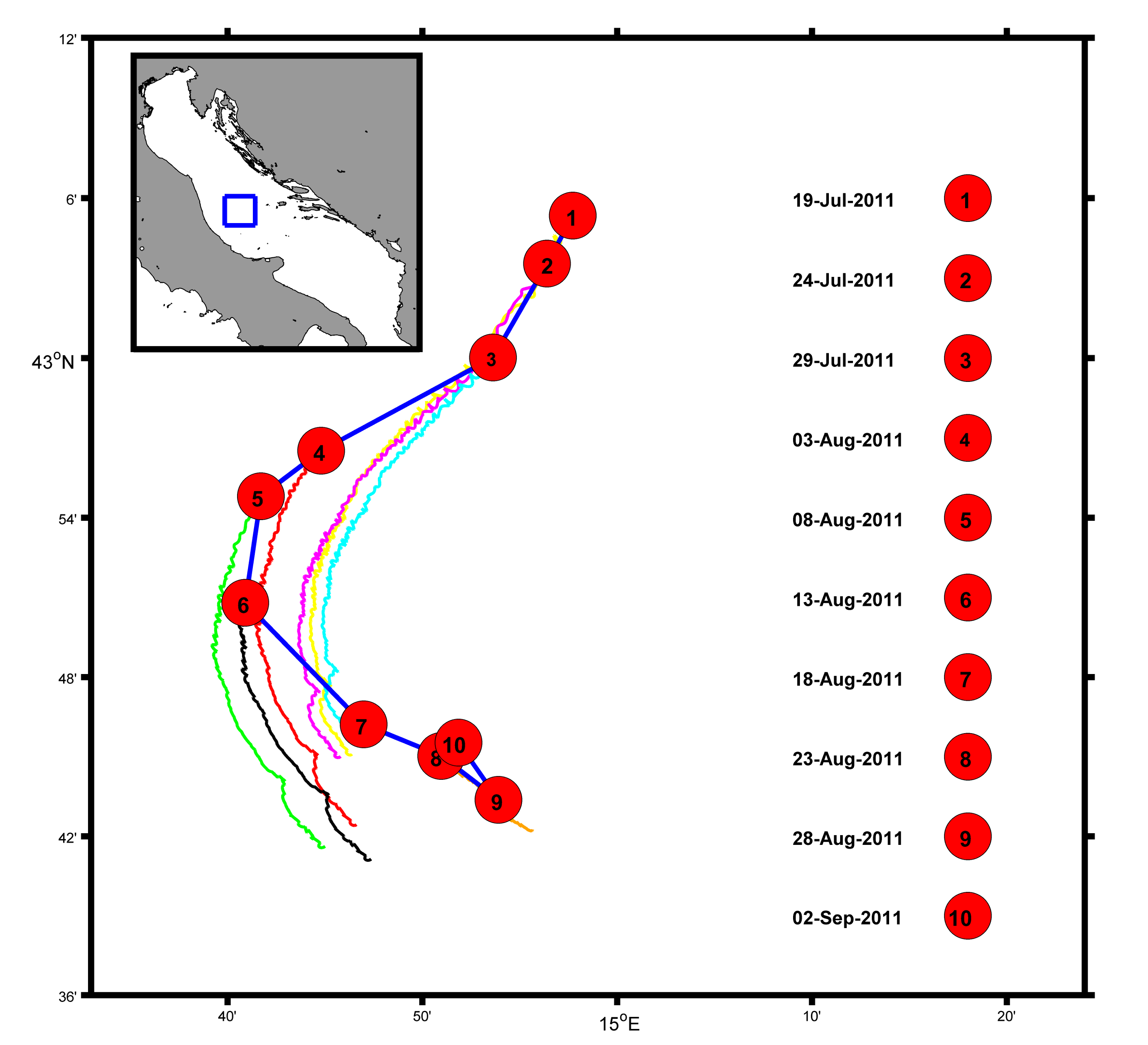


**Fig C. ROMS modelled (fuzzy lines) and measured drifter trajectories (blue straight lines) with positions (red circles) and dates of profiling.** Blue square on the Adriatic map (upper left corner) denotes area of the ARGO drifter movements.

References

Rixen M, Book JW, Cavanna JA, DART partners. Dynamics of the Adriatic in Real-Time - DART06A. DVD. NURC. La Spezia. 2006.

Dutour Sikirić MA., Janeković I, Kuzmić M. A new approach to bathymetry smoothing in sigma-coordinate ocean model. Ocean Modell. 2009; 29(2): 128-136.

doi:10.1016/j.ocemod.2009.03.009

Tudor M, Ivatek-Šahdan S, Stanešić A, Horvath K, Bajić A. Forecasting weather in Croatia using ALADIN numerical weather prediction model. In: Zhang Y, Ray P, editors. Climate Change and Regional/Local Responses pp. Rijeka (Croatia): InTech; 2013. pp. 59-88.

Raicich F. Notes on the flow rates of the Adriatic rivers. In: Technical Report RF 02/94. CNR Instituto Sperimentale Talassografico. Trieste. Italy. 1994. pp. 8.

Oddo P, Pinardi N, Zavatarelli M, Coluccelli A. The Adriatic Basin Forecasting System. Acta Adriat.2006; 47(Suppl.): 169–184.

Flather RA. A tidal model of the northwest European continental shelf. Mem Soc R Sci Liege. 1976; 6: 141–164.

Marchesiello P, McWilliams JC, Shchepetkin AF. Open boundary conditions for long term integration of regional oceanic models. Ocean Modell. 2001; 3: 1–20. [doi:10.1016/S1463-5003(00)00013-5](http://dx.doi.org/10.1016/S1463-5003%252800%252900013-5)
