# Supplementary figures and images for: Spillover of the Atlantic bluefin tuna offspring from cages in the Adriatic Sea: A multidisciplinary approach and assessment
Source: PLoS One. 2017 Nov 30;12(11):e0188956. doi: 10.1371/journal.pone.0188956 (PMC5708836; doi:10.1371/journal.pone.0188956)

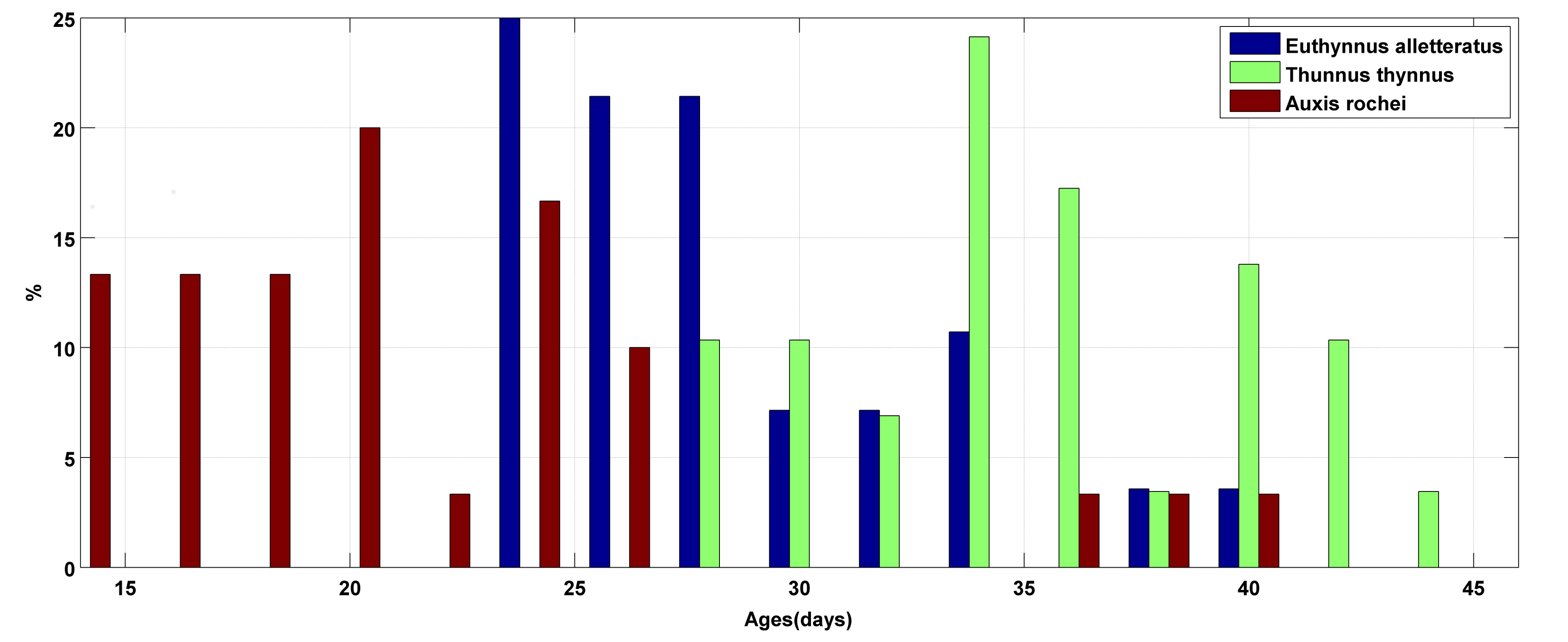

Supplement: S1 Fig — (TIF) [file pone.0188956.s004.tif]
